# Translational Selection Is Ubiquitous in Prokaryotes

**DOI:** 10.1371/journal.pgen.1001004

**Published:** 2010-06-24

**Authors:** Fran Supek, Nives Škunca, Jelena Repar, Kristian Vlahoviček, Tomislav Šmuc

**Affiliations:** 1 Division of Electronics, Rudjer Boskovic Institute, Zagreb, Croatia; 2 Division of Molecular Biology, Rudjer Boskovic Institute, Zagreb, Croatia; 3 Division of Biology, Faculty of Science, University of Zagreb, Zagreb, Croatia; 4 Department of Informatics, University of Oslo, Oslo, Norway; University of Arizona, United States of America

## Abstract

Codon usage bias in prokaryotic genomes is largely a consequence of background substitution patterns in DNA, but highly expressed genes may show a preference towards codons that enable more efficient and/or accurate translation. We introduce a novel approach based on supervised machine learning that detects effects of translational selection on genes, while controlling for local variation in nucleotide substitution patterns represented as sequence composition of intergenic DNA. A cornerstone of our method is a Random Forest classifier that outperformed previous distance measure-based approaches, such as the codon adaptation index, in the task of discerning the (highly expressed) ribosomal protein genes by their codon frequencies. Unlike previous reports, we show evidence that translational selection in prokaryotes is practically universal: in 460 of 461 examined microbial genomes, we find that a subset of genes shows a higher codon usage similarity to the ribosomal proteins than would be expected from the local sequence composition. These genes constitute a substantial part of the genome—between 5% and 33%, depending on genome size—while also exhibiting higher experimentally measured mRNA abundances and tending toward codons that match tRNA anticodons by canonical base pairing. Certain gene functional categories are generally enriched with, or depleted of codon-optimized genes, the trends of enrichment/depletion being conserved between Archaea and Bacteria. Prominent exceptions from these trends might indicate genes with alternative physiological roles; we speculate on specific examples related to detoxication of oxygen radicals and ammonia and to possible misannotations of asparaginyl–tRNA synthetases. Since the presence of codon optimizations on genes is a valid proxy for expression levels in fully sequenced genomes, we provide an example of an “adaptome” by highlighting gene functions with expression levels elevated specifically in thermophilic Bacteria and Archaea.

## Author Summary

Synonymous codons are not equally common in genomes. The main causes of unequal codon usage are varying nucleotide substitution patterns, as manifested in the wide range of genomic nucleotide compositions. However, since the first *E. coli* and yeast genes were sequenced, it became evident that there was also a bias towards codons that can be translated to protein faster and more accurately. This bias was stronger in highly expressed genes, and its driving force was termed translational selection. Researchers sought for effects of translational selection in microbial genomes as they became available, employing a flurry of mathematical approaches which sometimes led to contradictory conclusions. We introduce a sensitive and accurate machine learning-based methodology and find that highly expressed genes have a recognizable codon usage pattern in almost every bacterial and archaeal genome analyzed, even after accounting for large differences in background nucleotide composition. We also show that the gene functional category has a great bearing on whether that gene is subject to translational selection. Since presence of codon optimizations can be used as a purely sequence-derived proxy for expression levels, we can delineate “adaptomes” by relating predicted gene activity to organisms' phenotypes, which we demonstrate on genomes of temperature-resistant Bacteria and Archaea.

## Introduction

Due to non-random use of synonymous codons, protein coding sequences contain a layer of information on the DNA level that is not reflected at the protein sequence level. The principal determinant of codon usage in prokaryotes are nucleotide substitution patterns [1], [2] that vary greatly across genomes, as evidenced in the range of genomic G+C content spanned by the sequenced organisms. There is also significant variation in direction and strength of these nucleotide substitution biases along the prokaryotic chromosome [3] with a general tendency toward A+T-enrichment near the replication terminus. Another common intra-genomic trend in nucleotide composition concerns the distinction between the two DNA strands where the leading strand is ‘GC-skewed’, i.e. enriched in G over C and T over A [4] mostly due to deamination of cytosine in single-stranded DNA exposed during replication. Such biases in mutational processes may result from the nature of chemical changes to the nucleotides, but also from biases in errors of DNA replication and repair, and appear to be an important contribution to the background substitution patterns. In addition to the mutational biases, an adaptive component has also been proposed for specific nucleotide compositions, e.g. [5] and also for dinucleotides [6]. We refer the reader to a review of the organizational features of prokaryotic genomes with respect to local sequence composition and gene distribution [7].

In addition to the nucleotide substitution patterns, a competing influence on silent sites is selection acting to make protein translation more ‘efficient’ (in this context implying ‘faster’) and more accurate; although the term ‘efficiency’ is technically a misnomer [8], we use it for sake of consistency with previous literature. Traditionally, this effect was linked to abundances of tRNA isoacceptors for a particular codon [9], in agreement with a model where the speed of translational elongation is limited by availability of charged tRNA molecules [10]. Translational selection is also reflected in biased codon use that guards against missense and nonsense errors in proteins [11]. More recently, other more subtle translation-related determinants of codon usage have been observed, for instance the ‘load minimization’ where codons whose mutated forms cause less structural disruption to proteins are preferred [12] and the selective charging of tRNAs which promotes use of starvation-insensitive codons in amino acid biosynthetic pathways [13]. Some correlations have been observed between codon usage and protein structural features [14], and a synonymous mutation in a human gene was shown to produce a phenotype via an altered protein structure [15].

Selection for translational efficiency and accuracy would be expected to affect strongly a small set of highly abundant proteins, a typical representative being the ribosomal protein (RP) genes. The portion of a genome undergoing some degree of translational optimization may, however, be larger, and choice of genes within this subset was speculated to be related to the environment of a particular organism [16]. A number of prokaryotic genomes have been reported to show no influence of translational selection at all, most notably the slow-growing pathogens *Borrelia burgdorferi* [17] and *Helicobacter pylori* [18] or the insect endosymbionts *Buchnera* [19], *Wigglesworthia* [20] and *Blochmannia floridanus* [21]. However, in *Buchnera* a correlation was found between measured tRNA abundances and codon composition of highly expressed genes [22]. Three previous multiple-genome analyses detected evidence of translational selection in approx. 25% [23], 50% [24] or 70% [25] of the prokaryotes analyzed, the authors' conclusions depending heavily on the mathematical apparatus employed.

A multitude of statistics have been invented specifically for codon usage analyses and implemented in software [26], and many of these statistics can be generalized to measures of pairwise distances between codon frequency vectors. A prominent example is the popular ‘codon adaptation index’ [27] that measures the distance to a predefined set of highly expressed genes. Interestingly, several authors that relied on a codon distance measure have found that gene functions close in codon usage to RP genes in *E. coli* also have RP-like codon usage in some of the organisms which are supposed to lack translational selection, see e.g. the glycolysis genes in *H. pylori* [28] or respiration and ATP synthase genes in *B. floridanus* [29].

Correspondence analysis, an unsupervised dimensionality reduction technique followed by visualization or clustering, has often been used in single-genome studies to detect dominant trends in codon usage patterns. This approach has lead to qualitatively different results regarding presence or absence of translational selection depending on how the data was normalized, as demonstrated for *B. burgdorferi* [30], and for a larger number of genomes using a related technique of principal component analysis [31].

Our motivation for the present work was to reconcile the inconsistencies in the literature concerning the prevalence of translational selection among and within genomes, and its relationship to microbial ecology and physiology. To this end, we introduce a supervised machine learning-based computational framework that couples a classifier to standard statistical tests, an approach that exhibits an increased accuracy over commonly used unsupervised techniques, and the ability to control for a strong confounding factor – the nucleotide substitution patterns – that shape codon usage, but in a manner not related to protein translation.

## Results/Discussion

### The Random Forest classifier is an accurate method for codon usage analysis

**Figure 1:**
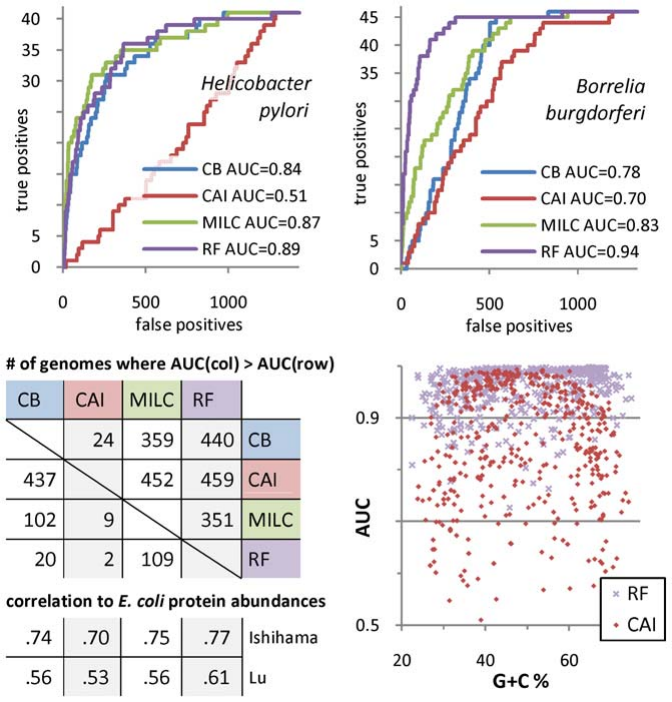
Comparison of methods for codon usage analysis. *Top left and right.* Performance of different classifiers utilizing codon frequencies in discriminating ribosomal protein genes from the rest of representative organism's protein genes. The receiver operating characteristic (ROC) curves show performance of: the Random Forest (RF) classifier [32], and the nearest centroid classifiers built around three distance measures of codon usage: CB, codon bias [33], CAI, codon adaptation index [27], and MILC, measure independent of length and composition [34]. *Bottom left.* Number of genomes (out of 461) where the column method outperforms the row method based on the area-under-ROC (AUC) statistic, and the rank correlation of the classifiers' per-gene class probabilities with experimental measurements of *E. coli* cytoplasmic protein abundances. All results were obtained in 4-fold crossvalidation. *Bottom right*. Dependence of AUC_CAI_ and AUC_RF_ on genomic G+C content; AUC_CAI_ is decreased in genomes with imbalanced G+C.

In contrast to previous approaches, we introduce a supervised machine learning-based framework for detecting the presence and the extent of translational selection in 461 prokaryotic genomes. Our method is based on the Random Forest (RF) classifier [32] which we evaluate in the task of discriminating a group of genes affected by selection for translational efficiency and/or accuracy, using only codon frequencies. The group of ribosomal protein (RP) genes is assumed to be highly expressed and therefore a representative subset of genes under such selective pressures; we back this assumption by a survey of RP mRNA abundances in a phylogenetically diverse set of organisms (Table S4). We demonstrate RF to be a more accurate tool (Figure 1) in comparison to three previous pairwise distance-based approaches [27], [33], [34]. Additionally, the RF predictions correlate with experimental measurements of protein concentrations in *Escherichia coli* slightly better than previous methods do (Figure 1, Table S1). We also show the widely used CAI method [27] to be suboptimal for genomes with imbalanced G+C content (Figure 1), as previously speculated by its author [35] and as evidenced by its inability to predict gene expression in the A+T rich eukaryote *Plasmodium falciparum* [34].

### Ribosomal protein genes have a distinctive codon usage not explained by underlying nucleotide substitution patterns

**Figure 2:**
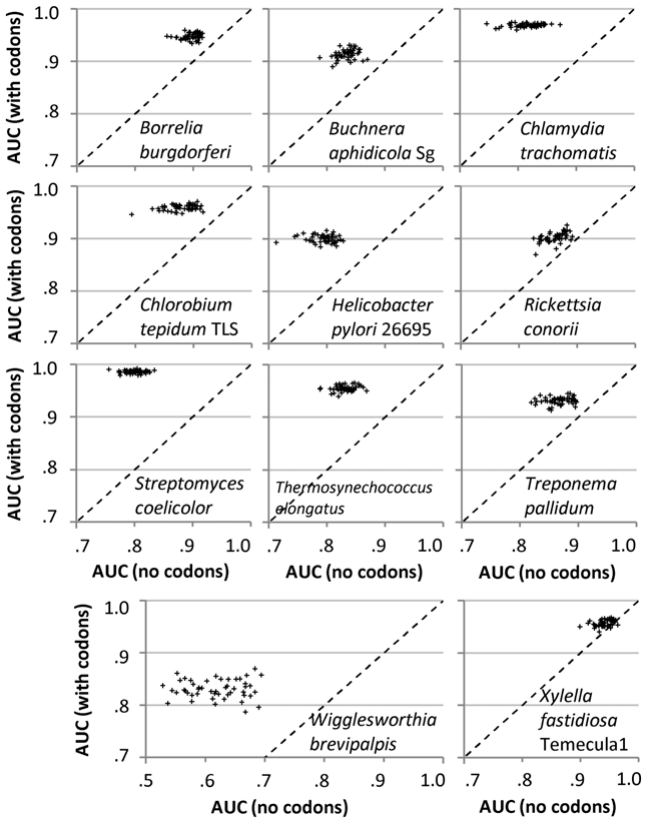
Predictive performance of the Random Forest classifier between datasets with and without codon frequencies. Performance is measured for the task of discriminating ribosomal protein genes from the rest of the protein coding genes, where each point represents a single run of four-fold crossvalidation. Points above the diagonal line signify improvement in AUC score with addition of codon frequencies, indicating that ribosomal protein genes have a characteristic pattern of codon usage which cannot be derived from the composition of intergenic DNA, a representation of the local nucleotide substitution patterns. The eleven genomes shown were cited as exhibiting no translational selection by each of the three previous multi-genome studies [23]–[25], see Text S1, Appendix B. Figure S2 shows the same experiments, but with codon frequencies shuffled between genes.

For each genome, we train two series of RF classifiers to discriminate ribosomal protein genes: first, a series of ‘baseline’ classifiers that have at their disposal the description of regional nucleotide substitution patterns as mono- and di-nucleotide frequencies in non-coding DNA in the genes' vicinity, followed by a second series of classifiers that introduces additional information about codon frequencies of genes (see Materials and Methods and Figure S1). In 460 of 461 examined genomes, the codon frequencies consistently facilitated classification over the baseline (Table S3), providing strong evidence that translational selection is, in fact, ubiquitous among prokaryotes. This trend also holds true in genomes which previous large scale studies [23]–[25] have found as lacking translational selection (Figure 2). The only genome where our method did not detect a translation-related codon bias was *Saccharophagus degradans* 2–40, although even this result may change depending on the size of the ‘window’ of ncDNA examined (Text S1). This genome was previously found to exhibit extensive mosaicism in G+C content [36], probably due to large amounts of recently horizontally transferred (HT) DNA which might not have had sufficient time to ‘ameliorate’ [37] to match the new host's translational apparatus. *S. degradans* genome serves as an example how strong local variation in background nucleotide frequencies, here caused by HT events, might obscure translation-related codon usage biases even if they did exist by boosting the accuracy of the baseline classifier; an empirical evaluation of influence of HT on our findings is given in Materials and Methods and in Text S2.

Between repetitions of the RF training on the same genome, accuracy of classifiers obtained using codon frequencies was also generally uncorrelated with the accuracy of baseline classifiers obtained without codon frequencies (Figure 2), further indicating that the codon usage brings into the datasets information independent of that encoded in intergenic DNA. The accuracies of codon-trained RF models also tend to deviate less between runs. On a side note, the unexpectedly strong RF accuracy obtained solely from the description of local nucleotide composition (Figure 2, Table S3) underscores the need to control for this confounding factor in codon usage analyses. It may prove fruitful to reinvestigate whether the so-called ‘genomic signatures’ – dinucleotide frequencies in DNA – are indeed invariant within bacterial and archaeal genomes, as claimed previously [38]. On the other hand, the strong RF accuracy that we observed with intergenic DNA might in significant part be attributed to the use of ncDNA windows that overlap for neighboring genes (see Materials and Methods).

Expectedly, the accuracy of codon-trained RF classifiers generally reflects the intensity of codon biases within a genome (Figure S3, Dataset S1), but the accuracy is also bound to be related to the proportion of genes within a genome that are affected by translational selection; see section below. The increase in accuracy for a genome additionally depends on the baseline model derived from local and between-strand variation in background nucleotide composition. Therefore, the magnitude of the increase does not have a straightforward interpretation in itself; rather, if the increase over the baseline has sufficient statistical support, it may be concluded that a translation-related codon usage bias is present, be it strong or weak.

### A sizeable portion of each genome shows an above-baseline codon usage similarity to the RP genes

**Figure 3:**
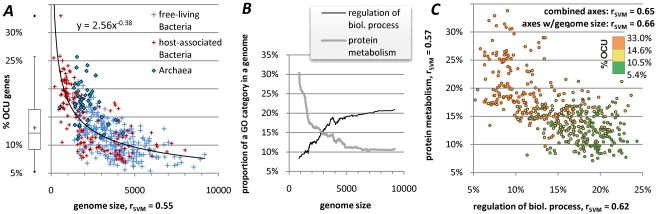
Extent of translational selection within genomes. (A) shows correlation of extent of translational selection in a genome (% OCU) to genome size, with the regression curve representing a fitted power-law relationship shown for illustrative purposes only. Genome size is expressed as number of protein coding genes at least 80 codons long. (B) shows the relationship between the genome size and “protein metabolism” and “regulation of biological process” functional categories, which is of predictable character; curves representing moving averages of the real data. (C) depicts correlation of % OCU to proportion of genes within a genome that belong to one of the two selected Gene Ontology categories from (B). “r_SVM_” referred to in (C) is the Pearson's correlation coefficient of a non-linear Support Vector Machines (SVM) regression fit (crossvalidation) of % OCU, for different combination of variables; values of “r_SVM_” obtained using one of the variables are given alongside the corresponding axis, top right inside the plot are values obtained when using both variables and in combination with the genome size.

The classifiers' predictions on a per-gene level provide estimates of similarity to the ribosomal protein genes. We declare a gene to have optimized codon usage (OCU) if this similarity exhibits a statistically significant increase after codon frequencies are introduced to the classifier (Figure 1). Using a conservative estimate (see Materials and Methods) we find that genomes contain on average 13.2% of OCU genes (Figure 3, Dataset S1), with a minimum of 5.4% in the metabolically versatile, free living *Pseudomonas flourescens* Pf-5, and a maximum of 33.0% in the highly reduced genome of the obligate parasite *Aster yellows witches-broom phytoplasma*. These estimates of the extent of translational selection within bacterial and archaeal genomes are broadly comparable to the results of a study in eukaryotes [39] that reported purifying selection at synonymous sites in ∼28% of the analyzed mouse-rat orthologs. Given these findings, it does not seem generally safe to assume that silent sites of all protein coding genes evolve neutrally, regardless of the domain of life under scrutiny.

The division of genes into the OCU and non-OCU groups does not imply that there is a clear-cut boundary between codon frequencies of the two groups. Rather, we would expect a gradient of codon usages to exist, where the genes labeled as OCU are those above the detection threshold of our method. This concept builds on an approach formulated by Karlin, Mrazek and colleagues [29], [33], [40] where a subset of genes in the genome is assigned the “PHX” (predicted highly expressed) label by codon usage similarity to a set of RP and other translation-related protein genes. There are, however, three important distinguishing features of OCU assignments: (a) they are based on a RF classifier that outperforms the ‘codon bias’ distance measure used for PHX assignments (Figure 1), (b) OCU is separated from non-OCU by a significance call of a statistical test instead of relying on an arbitrary threshold, and (c) OCU assignments are made using a control for local nucleotide substitution patterns which are a strong confounder in codon usage analyses.

In mammalian genomes, the presence of translationally selected codon usage is still an unresolved issue, see [41] for a recent analysis. The method we have here employed to prokaryotes could potentially be useful for future investigations on mammalian genomes where non-coding DNA is plentiful and local variation in GC content (isochores) greatly complicates analysis.

### Elevated mRNA expression levels of codon-optimized genes

**Figure 4:**
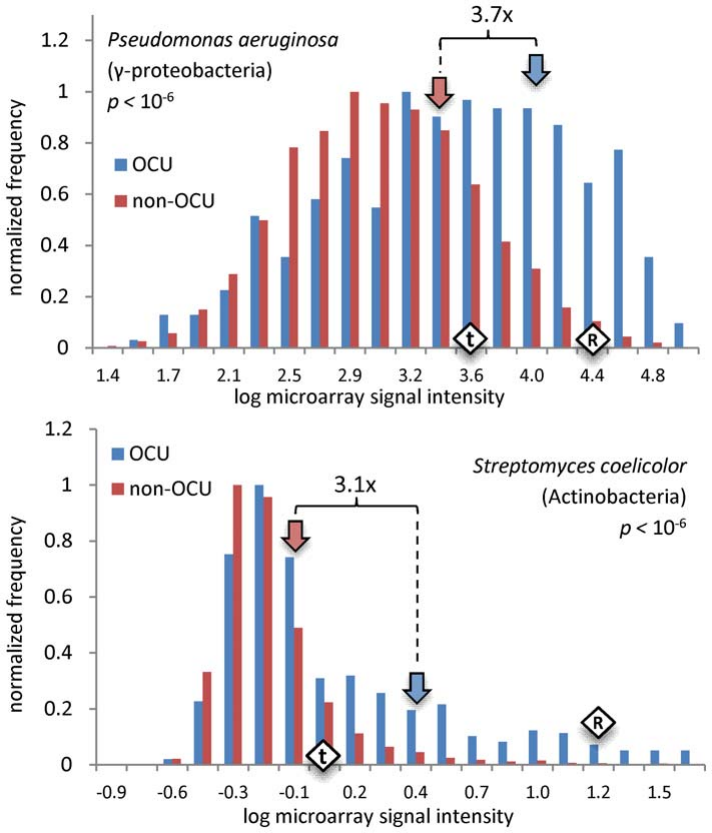
Expression levels of OCU versus non-OCU genes. Histograms comparing microarray signal intensities between genes with optimized codon usage (OCU) and the non-OCU genes. The *P. aeruginosa* and *S. coelicolor* genomes were previously considered to lack translational selection (Text S1, Appendix B). The *p*-values are by the Baumgartner-Weiss-Schindler permutation test [43]. Block arrows show the mean microarray signal intensity of OCU or non-OCU genes. Numbers above the curly braces are ratios of mean signal intensity of OCU genes to mean signal intensity of non-OCU genes. Diamonds show the mean signal intensity for aminoacyl-tRNA synthetases (“t”) or the ribosomal protein genes (“R”). Full data for 19 organisms in Table S5; average ratio of OCU expression to non-OCU expression in the 19 organisms is 2.4x. See Figure S4 for similar histograms, but with the ribosomal protein genes excluded.

We have demonstrated that in almost all examined genomes the RP genes can be discerned by their codon usage, even after local or strand-specific nucleotide composition are controlled for. To verify that this codon bias of the RP-like genes ( = OCU genes) is indeed due to translational selection, we examine the correlation of OCU/non-OCU assignments to gene expression data (Text S1, Appendix A) from 19 phylogenetically diverse species. We find that OCU genes record microarray signal intensities on average 2.4-fold higher than non-OCU genes (2.2x if RP genes are excluded), ranging from 1.2x to 3.7x; compare this to the 6.0x difference between RP – representing the most highly expressed genes – and the average measurement (Table S5). The ratio of means is significantly greater than unity in all genomes at *p*<0.01 (permutation test). Note that the relationship of the microarray signal intensities to mRNA abundances may be highly non-linear; in a study of gene expression in human tissues, the signal of Affymetrix microarrays was found to be roughly proportional with log-transformed counts of mRNA molecules obtained with Illumina sequencing [42]. In all 19 organisms we examined, the distribution of microarray signal intensities for OCU genes was significantly shifted towards higher values (*p*<0.01, Baumgartner-Weiss-Schindler permutation test [43]). The trend remains in the four of the 19 genomes where translational selection was previously considered to be ineffective (Figure 4, full data in Table S5). These estimates of correlation are likely conservative as gene expression will match codon usage better under certain growth conditions, presumably those that were dominant during the organism's evolutionary past [44]. It is not trivial to surmise these conditions in advance, and a dataset that matches them may not be available. Expression measurements taken under conditions of stress or starvation, for instance in the stationary phase, are expected to correlate less strongly with codon usage, as evidenced previously for *Bacillus subtilis* and *Escherichia coli* [34].

### Agreement of preferred codons with tRNA anticodons

**Figure 5:**
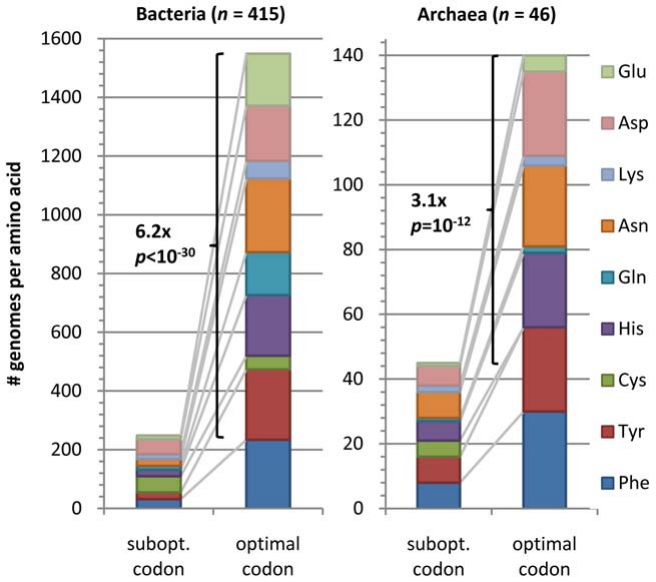
Preferred codons in OCU genes. Height of bar segments indicates the number of genomes in which the putatively translationally optimal or suboptimal codon is more frequent in the OCU genes *vs*. the non-OCU genes, broken down by amino acid. An optimal codon may be determined for a two-fold degenerate amino acid in cases when a genome codes only for tRNAs with one specific anticodon. The codon that directly matches this anticodon is then declared to be putatively optimal and is almost always C- or A-ending; the other codon is putatively suboptimal. Preference for a codon is determined by a Mann-Whitney U test on OCU vs. non-OCU codon frequencies at *p*<10^−3^. Shown *p* values are by sign test under the null hypothesis that OCU genes are equally likely to prefer optimal or suboptimal codons.

We looked for further evidence that OCU assignments were indeed due to translational selection by examining whether the OCU genes show a preference for putatively optimal codons in two-fold degenerate amino acids, where we assumed an optimal codon to match the tRNA anticodon by canonical base pairing (without wobble), as proposed in an early investigation of codon usage in yeast genes [45]. The presence or absence of tRNA genes with specific anticodons given in GtRNAdb database [46] indicates that the optimal codons in prokaryotes are almost always either C/A-ending, or undefined if the genome contains tRNA genes with both anticodons for the two-fold degenerate amino acid. We found that OCU genes prefer the putatively optimal codon 6.2x more frequently than the suboptimal one in Bacteria (*p*<10^−30^, sign test) and 3.1x more frequently in Archaea (*p* = 10^−12^, sign test; Figure 5; Table S6). All amino acids contribute approximately equally to this effect (Figure 5), with the exception of Cys which is rare and therefore hard to show a preference for or against, and Lys, for which an optimal codon cannot be defined in a majority of genomes. In spite of the dominant trend of OCU preference for optimal codons defined through tRNA content, some examples of amino acid-genome combinations where this regularity is reversed do exi*s*t; full data in Dataset S1. We speculate that these exceptions might stem from chemical modifications of nucleosides in the tRNA.

A rich structural diversity of the modifications exists in Bacteria and Archaea [47], many affecting the anticodon or its vicinity, thereby modulating the codon-anticodon interaction; reviewed in [48], [49]. The modifications prevent wobble matching to the wrong amino acid or enhance wobble matching to the correct amino acid, which may, in turn, render inapplicable the definition of cognate codons as the optimal codons. The potential for such modifications to elevate translation speed of ‘suboptimal’ codons over ‘optimal’ ones has been demonstrated experimentally on Drosophila tRNA^His^ [50] and on *E. coli* tRNA^Glu^ [51]. Currently, the tRNA nucleoside modifications are fully known only for few organisms [52]; it will be interesting to see how the future experimental data on the modifications will align with the OCU-preferred codons. Equally relevant to this issue is a recent paper discussing the choice of optimal codons in genomes [53], stating that optimal codons are dictated mainly by the direction of nucleotide substitution patterns evident in the overall genomic G+C content. Moreover, the authors have also found that in a large majority of bacterial genomes the gene set with the most highly biased codon usage is enriched with RPs and translation elongations factors, and that this codon usage cannot be reproduced from composition of intergenic DNA [53]. This finding is consistent with the notion of translationally selected codon usage as a prevalent phenomenon among prokaryotic genomes.

### Proportion of codon-optimized genes and genome size

**Figure 6:**
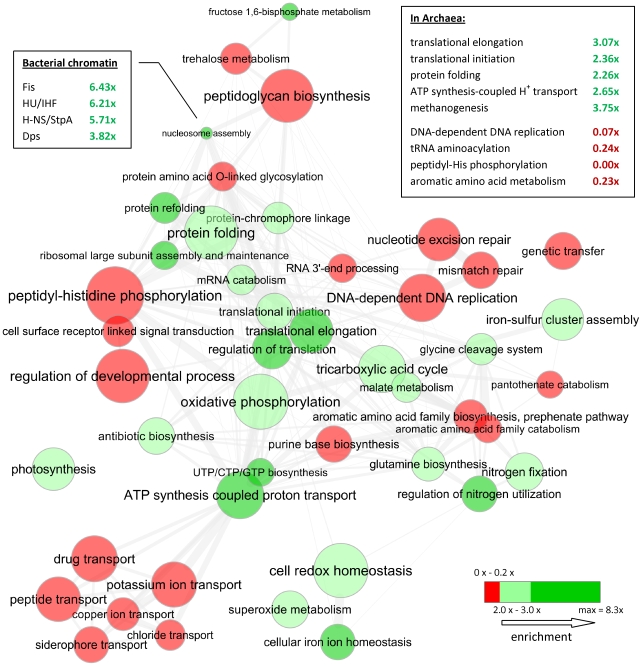
Gene ontology categories enriched with, or depleted of, OCU genes in Bacteria. Disc color indicates depletion (red) or enrichment (green), while size is proportional to log number of genes in category. Enrichment or depletion is significant at *p*<10^−15^ (Fisher's exact test) in all displayed categories. Thickness of grey lines represent semantic similarity between categories; also, spatial arrangement of discs approximately reflects a grouping of categories by semantic similarity. Displayed categories have been selected from a broader set to eliminate redundancy and prepared for visualization using the REViGO tool available at http://revigo.irb.hr/; see Dataset S2 for an exhaustive listing. Callout shows enrichment of selected orthologous groups within the “nucleosome assembly” category. Summary of results from Archaea is shown in the embedded frame.

The proportion of OCU genes correlates inversely to genome size (Figure 3A, Spearman's *ρ* = −0.71, Dataset S1), and we note a relationship of % OCU to lifestyle of bacteria, free-living vs. host-associated. These effects are largely a consequence of the changes in proportions of gene functional categories in genomes with regard to size (Figure 3B) [54], [55] and lifestyle, as the % OCU is readily predictable from frequencies of selected Gene Ontology (GO) categories (Dataset S1), even after controlling for genome size (Figure 3C). Note that our procedure to estimate of the extent of translational selection within genomes – expressed as % OCU genes – was not designed to measure the strength of this selection within a genome. Ideally, the genes' OCU assignments should be independent of the strength of the selection, previously recognized to differ greatly between genomes depending on the growth rate of the organism, or the composition of the cellular tRNA pool [56]. Therefore, the high % OCU in small genomes is not an indication that translational selection is stronger or weaker in these organisms, rather it is largely consequential to the proportion of underlying gene functional categories. If the extent of codon optimization within a genome is dictated mainly by its content of gene functions, one would expect the individual GO categories to have general preferences towards enrichment or depletion from OCU genes that are conserved across organisms, as we have verified (Figure 6, Table S7).

### Equivalent gene functional categories are optimized across domains of life

The most prominent trend in our results is consistent with previous research [28], [33], with translational selection readily acting on genes involved in protein production and in energy metabolism. Genes that are rarely OCU are involved in regulation, DNA replication and repair, sensing of stimuli, and most kinds of transport, except the electron transport chain and ATP synthesis-coupled H^+^ transport. Very similar trends are observed when focusing only on a subset of genomes previously cited as lacking translation-related codon usage biases (Table S8), corroborating the idea that translational selection is operative even in these genomes.

We compared Gene Ontology categories enriched with OCU genes between Bacteria and in Archaea (Figure 6, box), revealing a general agreement between the two domains of life. Two prior studies highlight an unusual reduction in frequency of codon-optimized ribosomal proteins as specific to Archaea [40] and also Archaea-specific optimizations in a DNA replication and repair protein PCNA [57] while our results do not support these observations. We suspect them to be artifacts of a methodology that does not control for the local variation in background nucleotide composition, coupled with the fact that in Bacteria – but not in Archaea – ribosomal protein genes are often collocated on the chromosome as they tend to share operon membership. Several eukaryotes have histone proteins which are highly biased towards the use of optimal codons [58]. Here, we report that Bacteria have nucleoid-associated proteins (Figure 6, callout) that are frequently OCU, identifying another instance of equivalent gene functions being translationally optimized in different domains of life. Furthermore, the archaeal chromatin protein AlbA is commonly OCU (40 out of 51 occurences, 4.6x enrichment, *p* = 10^−21^ by Fisher's exact test). These findings are supported by a quantitative proteomics experiment [59] that places the nucleoid-associated proteins Fis, H-NS and HU among the top 10 most abundant non-ribosomal proteins in the *E. coli* cytosol.

### Genes with codon usage contrasting the trend in their functional category

Aminoacyl tRNA synthetases (aa-tRS) are infrequently OCU (enrichment = 0.24x), consistent with their mRNA levels close to the genomic average (Table S4). A prominent exception is the Asn-tRNA charging enzyme (Table S9, enrichment = 1.05x), which might signal erroneous homology-based transfers of functional annotation involving some instances of this protein and the evolutionarily and structurally related [60] amino acid biosynthetic enzyme, asparagine synthetase A (AsnA, enrichment = 1.44x). A different NH_4_^+^-assimilating enzyme, glutamine synthetase, is also enriched with OCU genes (GlnA, enrichment = 2.12x), in contrast to a general avoidance of codon optimization in amino acid biosynthesis genes (enrichment = 0.50x). This leads us to speculate about an additional physiological role for GlnA and/or AsnA in Bacteria that would involve detoxification of ammonia by the energetically costly incorporation into amino acids, as has recently been demonstrated to occur in the yeast *S. cerevisiae* [61].

Aerobic respiration normally produces oxygen radical species which are then detoxified, or their damaging affects averted by scavenging of free iron, by an array of proteins that we found to be frequently OCU across Bacteria (Table S10). The abundance of proteins defending from oxidative stress therefore seems not to be specific to the radioresistant organism *Deinococcus radiodurans*, as claimed previously [33]. OCU genes are, however, conspicuously rare in the catalase genes in Bacteria (Table S10), possibly due to their activity being necessary only at supraphysiological levels of H_2_O_2_, as previous experimental work indicates is actually the case in *E. coli* [62].

### Lifestyle-specific adaptations in prokaryotes are reflected in codon optimizations

We have shown that a number of gene functional categories exhibit strong preferences toward or against translational optimization across all Bacteria and Archaea. If organisms defined by a specific lifestyle show a tendency contrary to the general trend, and this tendency is constrained to a gene functional category, this correlation could be biologically meaningful. An example illustrative of this principle was previously brought forward in work by Karlin [40] and Carbone [28], where the glycolysis genes were claimed to be more frequently codon-optimized in anaerobic bacteria. Indeed, we detected this association (GO∶6096, enrichment = 2.1x, *p* = 10^−33^) alongside with a – not unexpected – increase in OCU frequency of carbohydrate transporters (GO∶8643, enrichment = 1.8x, *p* = 10^−14^). We also found an increase in OCU frequency of ferritin in aerobes (COG∶1145, enrichment = 3.0x, *p* = 10^−17^), consistent with ferritin's role in protection against oxidative stress mediated by soluble ferrous ions.

In specific Archaea and Bacteria, the ability to thrive at high temperatures was expected to leave a distinct ‘signature’ in genome composition, implying that a gene complement responsible for this phenotype could be delineated. This is, however, generally not the case [54]. Perhaps the genome-encoded determinants of thermophily are discernible on a more fine-grained level, encompassing adaptation through changes in gene regulation and/or recruitment of existing genes for alternative physiological roles; let us name this set of alterations evolved in response to specific environmental challenges an ‘adaptome’ of an organism with respect to an environment. The hallmark feature of translational selection is that it affects highly expressed genes most strongly. The detection of codon optimizations on individual genes can thus be used as a proxy for the genes' expression level, offering an insight into adaptive changes in an organism's physiology; we would not expect the codon optimizations, as we measure them, to reflect other kinds of functional differences between proteins.

### An example of an “adaptome”: gene functions with optimized codon usage in thermophiles

Based on the correlations observed independently in Archaea and in Bacteria, we conjecture about two metabolic adjustments that would aid in protection of proteins and DNA against thermal denaturation. Phosphorylation is normally used to regulate protein activity, and regulatory proteins are typically not highly abundant in cells. Consequently, they were rarely labeled as OCU (Figure 6). However, genomes of thermophilic microbes tend to contain comparatively more OCU genes within the ‘protein phosphorylation’ functional category (Table 1) than the genomes of mesophiles. This difference would be explained if the addition of phosphate groups was a very commonly occurring process that affected a considerable fraction of total cellular protein, for instance if the phosphates served a structural role in many different proteins. Previous comparative analyses of structures of thermophile proteins versus their mesophile counterparts indicated that a typical characteristic of thermophile proteins is an increase of charged residues on the protein surface [63], [64]. Attaching charged phosphate groups (Table 1) to existing amino acids would lead to a similar effect, perhaps even to a stronger degree as the phosphate carries a higher charge than the side chains of charged amino acids. On the other hand, the activity level of such phosphorylation might be controlled through acetylation enzymes (Table 1) that would compete for the same substrates (amino acid side chains).

**Table 1:** Selected gene functions and groups of orthologous genes that are frequently translationally optimized in thermophilic Bacteria (*n* = 30) and Archaea (*n* = 27) in comparison to mesophilic Bacteria (*n* = 341) and Archaea (*n* = 17).

ID a	Description	Enrichment in Bacteria	Enrichment in Archaea
GO∶43687 ^b^	post-translational protein modification	2.77x, p = 10^−12^	6.16x, p = 10^−6^
GO∶16301 ^b^	kinase activity	-	2.90x, p = 10^−7^
GO∶4672 ^b^	protein kinase activity	4.84x, p = 10^−9^	(5.51x, p>10^−3^)
GO∶8080 ^b^	N-acetyltransferase activity	2.95x, p = 10^−15^	(2.11x, p>10^−3^)
GO∶45449 ^c^	regulation of transcription	2.18x, p = 10^−61^	2.48x, p = 10^−31^
GO∶156 ^c^	two-component response regulator activity	2.51x, p = 10^−18^	2.98x, p = 7·10^−4^
GO∶3677 ^c^	DNA binding	1.56x, p = 10^−27^	2.06x, p = 10^−24^
COG∶784 ^c^	CheY-like receiver	2.43x, p = 10^−10^	(2.69x, p>10^−3^)
COG∶640 ^c^	predicted transcriptional regulators	2.11x, p = 10^−9^	(1.66x, p>10^−3^)
COG∶1846 ^c^	transcriptional regulators	2.05x, p = 7·10^−4^	2.52x, p = 5·10^−4^
COG∶1595	DNA-directed RNA polymerase specialized sigma subunit, σ24 homolog (SigmaE)	2.53x, p = 10^−5^	(COG absent from Archaea)
COG∶1708	predicted nucleotidyltransferases	2.27x, p = 10^−5^	4.38x, p = 10^−5^

a Prefix “GO” denotes a Gene Ontology [78] category; “COG” denotes a group from the Clusters of Orthologous Groups database [79].

b, c Used to infer putative mechanisms of protein protection (b), or DNA protection (c) in thermophiles.

A putative DNA thermoprotective mechanism could be inferred from an unexpectedly high frequency of codon optimization within thermophile genes annotated as response regulators for two-component systems and other transcriptional regulators. Again, we would generally not expect high expression levels from regulatory proteins, unless they were to perform a different role in the cell, either alone or in addition to their original function. We speculate that a subset of these proteins with DNA binding domains (candidates in Table 1) might play a role in formation of a chromatin-like structure that would act to preserve DNA geometric properties, protect it from chemical damage or aid in repair under high temperatures. To our knowledge, there is currently no experimental data that would directly support this hypothesis; however, on the other end of the temperature spectrum, stabilization of DNA and RNA secondary structures occurs and is known to be counteracted by overproduction of nucleic acid binding proteins and RNA helicases when mesophiles are brought into cold conditions [65]. Our results indeed show that psychrophilic Bacteria have increased translational optimization of genes with ATP-dependent helicase activity (GO∶8026, enrichment = 3.1x, *p* = 10^−4^; Dataset S2).

We have performed numerous other statistical comparisons involving 35 distinct microbial lifestyles or phenotypes and information on genes' codon optimizations within functional categories. An exhaustive listing of significant results is available in Dataset S2 and on the authors' website at http://www.adaptome.org; here, care should be taken in interpretation as the lifestyles/phenotypes are intercorrelated and also not independent from phylogenetic subdivisions. We hope this data will stimulate further research and help direct experimental work to elucidate environmental adaptations of microbes. Moreover, since the ‘adaptomes’ are purely sequence-derived, an equivalent – or improved – computational methodology can be applied to genomes as soon as they are sequenced, cutting time and effort required to understand the physiology of novel organisms.

## Materials and Methods

### Prokaryotic genomes and construction of datasets

We have downloaded 621 fully sequenced prokaryotic genomes from the NCBI Entrez Genome FTP site [66] and removed multiple strains of a single species to retain the strain best covered by Gene Ontology annotations, leaving 461 genomes. Information about lifestyles of organisms was assembled from the JCVI Genome Properties [67], and the NCBI Entrez Microbial Genome Properties [68], and curated. The original data used in all computations is freely available via the authors' web site at http://www.adaptome.org/.

To construct datasets – one per genome – we declare all genes coding for ribosomal proteins (RP) to be the ‘positive class’, including the rare cases (approx. 0.6 occurences per genome) of multicopy RP genes. All other protein-coding genes are the ‘negative class’. Genes shorter than 80 codons were excluded from computation. A gene is represented by a series of codon frequencies for all degenerate codon families (excluding the stop codons) where the frequencies of codons for a single amino acid are normalized to add up to one. Codon frequencies of amino acids absent from a protein are coded by a ‘missing value’ symbol.

### Comparing a classifier to measures of codon usage

Ten-fold crossvalidation was run to determine performance of the Random Forest (RF) classifier in discriminating the RP genes by their codon frequencies; the area-under-ROC-curve (AUC) score [69] was recorded. The AUC is a measure independent of class sizes that ranges from 0.5 for a random classification model to 1.0 for a perfect model. The RF algorithm [32] produces an ensemble of decision tree classifiers, where each decision tree is constructed by recursively partitioning the data by attribute value tests (forming ‘nodes’) so as to reduce the entropy of the class label in the resulting partitions (‘branches’). In RF, trees are constructed on bootstrap samples of the entire dataset, and choice of attributes at each node is restricted to introduce variability. The final predictions of a RF model are obtained by averaging over individual trees (‘voting’). Regarding the specific implementation of the RF algorithm, we used FastRandomForest [70].

RF was compared to three pairwise distance measures for vectors of codon frequencies: (i) the “codon bias between gene groups” (CB) is essentially a weighted Manhattan distance employed by Karlin and colleagues [33] for finding ‘predicted highly expressed’ genes in microbial genomes; (ii) the “codon adaptation index” (CAI) is an established surrogate for gene expression under optimal growth conditions of *Escherichia coli* and *Saccharomyces cerevisiae* [27]; and (iii) the “measure independent of length and composition” (MILC) [34] is a corrected χ^2^-type statistic devised to address methodological deficiencies in other approaches such as CB. For a thorough description and formulae for calculation of these measures, see Text S1.

We have incorporated the three distance measures into a ‘nearest class centroid’-type classifier, analogous to uses of CB and CAI in the literature, and compared AUC scores of the RF classifier to the nearest centroid classifiers (Figure 1). As another verification of the RF classifier, we have compiled protein abundance data in the *E. coli* cytoplasm from two quantitative proteomics experiments, Ishihama *et al*. [59] and Lu *et al*. [71]. After retaining data for 369 proteins that occur in both studies, we computed Spearman's rank correlation of the methods' output (probability of belonging to positive class, in crossvalidation), and protein abundances (Figure 1); also the correlations with the full experimental data are given in Table S1.

### Detecting translational selection in genomes

We encode the information about nucleotide substitution patterns underlying the sequence of each gene by computing mononucleotide and dinucleotide frequencies in the non-coding regions of DNA neighboring the translated part of the gene. Genes for functional RNA molecules such as tRNA and rRNA are also treated as coding DNA and thus do not contribute toward composition of non-coding (intergenic) DNA. The size of the neighborhood window was set to either 5, 10 or 20 kilobases upstream from the gene's start codon, and 5, 10 or 20 kilobases downstream from the stop codon. The window size of 10 kb upstream +10 kb downstream guarantees that in 99% of the genomes (457 out of 461), 99% of the genes have at least 142 non-coding nucleotides available for estimation of nucleotide substitution patterns (Table S2). To detect if translational selection acts on a genome, the RF classifier is first trained to distinguish RP genes (‘positive class’) based solely on the mono- and di-nucleotide frequencies of genes' neighboring non-coding DNA within a given window size. Fifty runs of four-fold crossvalidation are used to estimate the accuracy of the classifier, and the AUC score for each of the 50 runs is recorded. The crossvalidation is stratified, meaning that genes are sampled so that the proportions of the RP genes are conserved in the training and the testing parts of each crossvalidation split. The 50 runs of crossvalidation are then repeated for a second time, however now the codon frequencies are also included in the dataset for the RF classifier training, in addition to description of the intergenic regions. The sign test [72] is used to compare AUC scores obtained without codon frequencies to AUC scores obtained with codon frequencies, for each genome (Figure S1). A summary of results for window size 10k is presented in Table S3.

Note that the described approach to determine mono/di-nucleotide frequencies implies that for neighboring genes the corresponding ncDNA windows will overlap, meaning the description of background nucleotide composition will not be independent between the neighbors. Consequently, the estimate of crossvalidation accuracy (AUC) will be somewhat optimistic for the baseline, ncDNA-only models. This should, however, not be problematic as our conclusions regarding presence of translational selection can be biased only to the conservative side due to this issue – having ncDNA models of higher AUC means it can be only more difficult to surpass the models' AUC using the ncDNA+codon models.

### Assigning optimized codon usage (OCU) labels to individual genes

During the procedure described above which involves two rounds of RF classifier training – without and with codon frequencies – the per-gene probabilities of belonging to positive class are recorded for each of the 50 runs of crossvalidation, and compared between the two rounds of crossvalidation (Figure S1). A sign test [72] is used to determine if an increase in probability occurs more frequently than expected by chance; if it does, the gene is labeled as having optimized codon usage (OCU). At this point, we combine the OCU assignments obtained with the three values of the window size parameter (5, 10 and 20 kilobases) into a consensus set by determining the median *p*-value of sign tests of the three window sizes for each gene. The agreement between window sizes is analyzed in Text S2. The *p*-values of OCU assignments for each gene are available as Dataset S1 and from the authors' website at http://www.adaptome.org. Additionally, the full Java source code that performs all calculations described in the Materials and Methods section will be made freely available from the same website in the near future, or on request from the authors.

We have set the default *p*-value threshold to 10^−15^, corresponding to exactly 50 out of 50 sign test ‘wins’ for the dataset with codon frequencies. This sign test *p*-value should be regarded as somewhat optimistic because the repeated runs of crossvalidation are not independent, being based on repeated sampling from the same set of genes. To obtain a conservative estimate, we employed a corrected paired *t*-test [73] intended for comparison of classification algorithms using repeated runs of crossvalidation. Note that we here compare RF models derived from a specific dataset, and not the different variants of the underlying RF algorithm itself, and therefore we would expect the *p*-value obtained with this test to be pessimistic for our experimental setup [73]. After this corrected *t*-test, the median *p*-value for OCU genes in 10 representative genomes (Text S2) was 6·10^−6^, while for 95% of the OCU genes, *p*<2·10^−3^. When testing for difference of AUC scores with/without codons, the median *p*-value for all 461 genomes obtained by the corrected paired *t*-test was *p* = 10^−13^; for 95% of the genomes, *p*<4·10^−5^ (compare to median *p* = 10^-15^ by sign test).

### Testing robustness of OCU assignments

We test the robustness of the genes' OCU assignments by performing several computational experiments. First experiment is a simulation to see if a methodological bias exists where changes in the positive-to-negative class size ratio would affect frequency of OCU labels; we demonstrate the extent of such changes to be minor (Text S2) and insufficient to explain the anticorrelation between genome size and % OCU described in Results (Figure 3A). The second experiment verifies if an outlier in the positive class – a RP gene with atypical codon usage – would affect OCU assignments. Our RF-classifier based methodology for OCU detection was remarkably robust (Text S2) to such errors in annotation that might stem from e.g. pseudogenes or from the uncommon occurrence of RP gene horizontal transfer [74].

The third issue concerns non-coding DNA, which is generally not abundant in prokaryotes and therefore non-negligible parts of it may be occupied by regulatory elements subject to selection. This should have little impact on our results, as our representation of the ncDNA is not complex enough to permit distinction between ncDNA containing specific regulatory motifs, unless the motifs were to differ strongly in their overall composition and occupy the major part of the ncDNA. Even if this were the case, our procedure would err on the conservative side, leading to an overestimation of the influence of nucleotide substitution patterns and consequentially a lower % OCU, and only if there was a correlation between the composition of regulatory regions and expression levels. We have empirically verified to what extent selection on ncDNA affects our results by re-running the computational framework (Figure S1) on a subset of genomes while excluding the ncDNA regions 20 bp upstream of the translation start codon; this region was shown to be under much stronger selection than the rest of the prokaryotic ncDNA [75] as it contains translation-related sequence elements. The OCU assignments were sufficiently robust to this perturbation (Text S2) as the resultant changes are commensurate to the variability of the method itself.

A fourth experiment concerns the influence of putatively horizontally transferred (HT) regions on our results. Our method will treat a HT region in the same as it treats the regions that differ due to (endogenous) local or strand-specific variation in background nucleotide composition, i.e. regardless of the biological mechanism that causes a deviation in the background nucleotide frequencies, we aim to detect shifts away from this background and toward RP-like codon usage. We empirically evaluated the influence of HT on our results by taking 10 representative genomes and masking the segments marked as HT in the IslandViewer database [76] by any of the three underlying HT-detection algorithms to have highest possible coverage. Then we repeated the OCU finding procedure and compared assignments to the original ones (Text S2). Again, the changes induced by this filtering were generally commensurate to the between-run variability of the method, save for a slight increase in %OCU in several genomes, meaning the original estimate of %OCU may be conservative with regard to horizontal gene transfer.

### Tests of enrichment for optimized genes and display of results

We use a series of Fisher's exact tests for association of two categorical variables [77] to describe distribution of OCU genes along Gene Ontology [78] categories or COG orthologous groups [79] in all analyzed genomes, and in genomes grouped by environmental and phenotypic contexts (lifestyles). We perform two kinds of tests: ‘Test A’ operates across all genes in all genomes, and compares distribution of optimized genes within a GO category/COG group to the distribution of optimized genes outside the GO/COG, and is iterated over all GOs/COGs; ‘Test B’ operates within a single GO category/COG group, and compares organisms with a specific lifestyle to all organisms which are known not to possess the lifestyle. ‘Test B’ is iterated over all possible combinations of GO/COG and lifestyle; we discuss only the thermophilic lifestyle in the manuscript, while data on other lifestyles is given in Dataset S2. We set the threshold *p*-value to 10^−3^, yielding a 1.6% false discovery rate among the GO categories/COGs found to be enriched or depleted in OCU genes in Archaea, and a false discovery rate of 1.7% in Bacteria. All tests passing these criteria were additionally screened to retain only tests with a sufficient magnitude of enrichment or depletion of optimized genes (>1.50x or <0.67x) between GO categories/COGs (test A) or organism groups (test B). All test results passing the thresholds for gene group size, statistical significance and magnitude of enrichment/depletion are available in Dataset S2 and from the authors' Web site http://www.adaptome.org/. Filtered subsets of the GO categories with reduced redundancy are also available as a part of Dataset S2; they were prepared using the REViGO tool available at http://revigo.irb.hr/.

A more detailed description of the employed computational methods and procedures is provided in Text S1 and summarized in a flowchart diagram in Figure S1.

## Supporting Information

Dataset S1 OCU/non-OCU gene calls in 461 Bacterial and Archaeal genomes, and summary tables of genome properties and OCU preferences for specific codons.
(4.77 MB ZIP)

Dataset S2 Tests for enrichment or depletion of OCU genes in Gene Ontology functional categories and COG orthologous groups.
(0.38 MB ZIP)

Figure S1 The workflow of the computational framework for detecting translational selection. Cylinders represent databases, rectangles represent operations and/or computation, parallelograms represent datasets, rounded rectangles within dotted frames represent endpoints, circles are references to Figures and Tables.
(2.72 MB TIF)

Figure S2 Predictive performance of the Random Forest classifier on datasets with codon frequencies permuted between genes. All datasets contain (non-permuted) attributes with mono- and di-nucleotide frequencies. This figure is analogous to Figure 2 from the manuscript and is intended to demonstrate that an increase in classifier AUC evident in Figure 2 cannot result purely from the addition of extra attributes if these attributes carry no useful information.
(0.22 MB TIF)

Figure S3 Correlation of the crossvalidation accuracy of RF classifiers with an estimate of intensity of genome-wide codon biases. Accuracy (as AUC score) of RF models trained on composition of intergenic DNA (top) and on composition of intergenic DNA plus codon frequencies (below) is compared to the MILC measure of distance between codon frequencies of ribosomal protein genes, and the rest of the genes within a genome.
(0.82 MB TIF)

Figure S4 Expression levels of OCU versus non-OCU genes, with ribosomal protein genes excluded. Histograms show microarray signal intensities for OCU and non-OCU genes in *P. aeruginosa* and *S. coelicolor* after removing the ribosomal protein genes from the genomes. This figure is analogous to Figure 4 in all other aspects.
(0.23 MB TIF)

Table S1 Correlations of *E. coli* cytoplasmic protein abundances with per-gene class probabilities of Random Forest and three codon distance measures. “Overlapping data” implies that only the genes present in both studies were considered. The Pearson correlation was computed after removing two proteins with extremely high abundance values in the Ishihama dataset.
(0.04 MB DOC)

Table S2 A survey of the amount of non-coding DNA within bacterial and archaeal genomes. Alongside the data for ten representative genomes, two additional rows display: the median values for the entire set of 461 genomes, and the 1^st^ percentile of values for the 461 genomes. Table cells show the number of non-coding nucleotides in a window size of 10 kilobases upstream of a gene's start codon, and 10 kilobases downstream of the stop codon.
(0.04 MB DOC)

Table S3 Accuracy of the RF classifier in the task of discriminating ribosomal protein genes. Accuracy is expressed as area-under-ROC-curve (AUC) score, and given for RF classifiers trained without codon frequencies (“AUC, no codons”) and with codon frequencies (“AUC, with codons”). Mean and standard deviation of AUC are computed from 50 runs of crossvalidation. The sign test p-value indicates whether the AUC score exhibits a statistically significant increase with introduction of codon frequencies to the classifier. Ten representative genomes are shown, along with three genomes with the least significant p-values among all 461 genomes. Full data is available as Dataset S1, or from the website http://www.adaptome.org/.
(0.05 MB DOC)

Table S4 Microarray signal intensities for ribosomal protein genes and aminoacyl-tRNA synthetases (aa-tRS). See Text S1, Appendix 1 for detailed information of which NCBI GEO Samples were chosen from the NCBI GEO Series in the table.
(0.05 MB DOC)

Table S5 Differences in microarray signal intensities for OCU and non-OCU genes. “BWS” stands for Baumgartner-Weiss-Schindler permutation test used to determine if the distribution of microarray signal intensities of OCU genes is shifted in comparison to the distribution of microarray signal intensities of non-OCU genes.
(0.05 MB DOC)

Table S6 Preferences of OCU genes towards optimal or sub-optimal codons for two-fold amino acids, as defined by the genome's tRNA gene content. Table cells show the number of genomes where OCU genes prefer the optimal codon, the suboptimal codon, or where there is no preference towards either codon. Preference for codons is detected by the Mann-Whitney U test on codon frequencies of OCU versus non-OCU genes, at *p*<10^−3^. Optimal codons are those directly recognized by the anticodon of a tRNA encoded in the genome [1], and the suboptimal codons, conversely, have no tRNA with the appropriate anticodon. In some cases (frequently for Lys, Gln and Glu), genomes may encode tRNAs with both anticodons and the optimal/suboptimal anticodon cannot be defined; therefore the “sum” column may be lesser than the total number of genomes.
(0.04 MB DOC)

Table S7 Protein functional categories enriched with (or depleted of) OCU genes in the “molecular function” namespace of the Gene Ontology. The list is filtered to exclude a number of categories redundant to the ones displayed using the REViGO tool available at http://revigo.irb.hr/; for a complete listing, please refer to Dataset S2, or the authors' website at http://www.adaptome.org
(0.16 MB DOC)

Table S8 A selection of protein functional categories enriched with (or depleted of) OCU genes in the “biological process” namespace of the Gene Ontology. This table is derived only from 38 organisms whose genomes were claimed to lack translational selection in at least 2 of 3 previous large-scale studies, see Appendix A in Text S1 for listing of the 38 genomes. Data in this table is analogous to the data in Figure 6 of the manuscript in all other aspects except for the restricted choice of genomes in this table.
(0.07 MB DOC)

Table S9 Depletion of OCU genes within the aminoacyl-tRNA synthetases in Bacteria.
(0.06 MB DOC)

Table S10 Enrichment and depletion of OCU genes within COG groups related to defense from oxidative stress. IDs in the form “COG:xxxx” denote groups from the Clusters of Orthologous Genes database.
(0.05 MB DOC)

Text S1 Supporting methods.
(0.15 MB DOC)

Text S2 Tests of robustness of the RF classifier-based methodology for assigning OCU labels to genes.
(0.17 MB DOC)

## References

[1] Chen SL, Lee W, Hottes AK, Shapiro L, McAdams HH (2004). Codon usage between genomes is constrained by genome-wide mutational processes.. Proc Natl Acad Sci U S A.

[2] Knight RD, Freeland SJ, Landweber LF (2001). A simple model based on mutation and selection explains trends in codon and amino-acid usage and GC composition within and across genomes.. Genome Biol.

[3] Daubin V, Perriere G (2003). G+C3 structuring along the genome: a common feature in prokaryotes.. Mol Biol Evol.

[4] Lobry JR, Sueoka N (2002). Asymmetric directional mutation pressures in bacteria.. Genome Biol.

[5] Rocha EP, Danchin A (2002). Base composition bias might result from competition for metabolic resources.. Trends Genet.

[6] Zeldovich KB, Berezovsky IN, Shakhnovich EI (2007). Protein and DNA sequence determinants of thermophilic adaptation.. PLoS Comput Biol.

[7] Rocha EP (2004). The replication-related organization of bacterial genomes.. Microbiology.

[8] Dethlefsen L, Schmidt TM (2005). Differences in codon bias cannot explain differences in translational power among microbes.. BMC Bioinformatics.

[9] Kanaya S, Yamada Y, Kudo Y, Ikemura T (1999). Studies of codon usage and tRNA genes of 18 unicellular organisms and quantification of Bacillus subtilis tRNAs: gene expression level and species-specific diversity of codon usage based on multivariate analysis.. Gene.

[10] Xia X (1998). How optimized is the translational machinery in Escherichia coli, Salmonella typhimurium and Saccharomyces cerevisiae?. Genetics.

[11] Stoletzki N, Eyre-Walker A (2007). Synonymous codon usage in Escherichia coli: selection for translational accuracy.. Mol Biol Evol.

[12] Najafabadi HS, Lehmann J, Omidi M (2007). Error minimization explains the codon usage of highly expressed genes in Escherichia coli.. Gene.

[13] Dittmar KA, Sorensen MA, Elf J, Ehrenberg M, Pan T (2005). Selective charging of tRNA isoacceptors induced by amino-acid starvation.. EMBO Rep.

[14] Oresic M, Shalloway D (1998). Specific correlations between relative synonymous codon usage and protein secondary structure.. J Mol Biol.

[15] Kimchi-Sarfaty C, Oh JM, Kim IW, Sauna ZE, Calcagno AM (2007). A “silent” polymorphism in the MDR1 gene changes substrate specificity.. Science.

[16] Carbone A (2006). Computational prediction of genomic functional cores specific to different microbes.. J Mol Evol.

[17] McInerney JO (1998). Replicational and transcriptional selection on codon usage in Borrelia burgdorferi.. Proc Natl Acad Sci U S A.

[18] Lafay B, Atherton JC, Sharp PM (2000). Absence of translationally selected synonymous codon usage bias in Helicobacter pylori.. Microbiology.

[19] Rispe C, Delmotte F, van Ham RC, Moya A (2004). Mutational and selective pressures on codon and amino acid usage in Buchnera, endosymbiotic bacteria of aphids.. Genome Res.

[20] Herbeck JT, Wall DP, Wernegreen JJ (2003). Gene expression level influences amino acid usage, but not codon usage, in the tsetse fly endosymbiont Wigglesworthia.. Microbiology.

[21] Banerjee T, Basak S, Gupta SK, Ghosh TC (2004). Evolutionary forces in shaping the codon and amino acid usages in Blochmannia floridanus.. J Biomol Struct Dyn.

[22] Charles H, Calevro F, Vinuelas J, Fayard JM, Rahbe Y (2006). Codon usage bias and tRNA over-expression in Buchnera aphidicola after aromatic amino acid nutritional stress on its host Acyrthosiphon pisum.. Nucleic Acids Res.

[23] dos Reis M, Savva R, Wernisch L (2004). Solving the riddle of codon usage preferences: a test for translational selection.. Nucleic Acids Res.

[24] Carbone A, Kepes F, Zinovyev A (2005). Codon bias signatures, organization of microorganisms in codon space, and lifestyle.. Mol Biol Evol.

[25] Sharp PM, Bailes E, Grocock RJ, Peden JF, Sockett RE (2005). Variation in the strength of selected codon usage bias among bacteria.. Nucleic Acids Res.

[26] Supek F, Vlahovicek K (2004). INCA: synonymous codon usage analysis and clustering by means of self-organizing map.. Bioinformatics.

[27] Sharp PM, Li WH (1987). The codon Adaptation Index–a measure of directional synonymous codon usage bias, and its potential applications.. Nucleic Acids Res.

[28] Carbone A, Madden R (2005). Insights on the evolution of metabolic networks of unicellular translationally biased organisms from transcriptomic data and sequence analysis.. J Mol Evol.

[29] Mrazek J, Spormann AM, Karlin S (2006). Genomic comparisons among gamma-proteobacteria.. Environ Microbiol.

[30] Perriere G, Thioulouse J (2002). Use and misuse of correspondence analysis in codon usage studies.. Nucleic Acids Res.

[31] Suzuki H, Saito R, Tomita M (2005). A problem in multivariate analysis of codon usage data and a possible solution.. FEBS Lett.

[32] Breiman L (2001). Random forests.. Machine Learning.

[33] Karlin S, Mrazek J (2000). Predicted highly expressed genes of diverse prokaryotic genomes.. J Bacteriol.

[34] Supek F, Vlahovicek K (2005). Comparison of codon usage measures and their applicability in prediction of microbial gene expressivity.. BMC Bioinformatics.

[35] Grocock RJ, Sharp PM (2002). Synonymous codon usage in Pseudomonas aeruginosa PA01.. Gene.

[36] Weiner RM, Taylor LE, Henrissat B, Hauser L, Land M (2008). Complete genome sequence of the complex carbohydrate-degrading marine bacterium, Saccharophagus degradans strain 2-40 T.. PLoS Genet.

[37] Lawrence JG, Ochman H (1997). Amelioration of bacterial genomes: rates of change and exchange.. J Mol Evol.

[38] Karlin S (1998). Global dinucleotide signatures and analysis of genomic heterogeneity.. Curr Opin Microbiol.

[39] Resch AM, Carmel L, Marino-Ramirez L, Ogurtsov AY, Shabalina SA (2007). Widespread positive selection in synonymous sites of mammalian genes.. Mol Biol Evol.

[40] Karlin S, Brocchieri L, Campbell A, Cyert M, Mrazek J (2005). Genomic and proteomic comparisons between bacterial and archaeal genomes and related comparisons with the yeast and fly genomes.. Proc Natl Acad Sci U S A.

[41] Parmley JL, Huynen MA (2009). Clustering of codons with rare cognate tRNAs in human genes suggests an extra level of expression regulation.. PLoS Genet.

[42] Marioni JC, Mason CE, Mane SM, Stephens M, Gilad Y (2008). RNA-seq: an assessment of technical reproducibility and comparison with gene expression arrays.. Genome Res.

[43] Neuhauser M, Senske R (2004). The Baumgartner-Weiss-Schindler test for the detection of differentially expressed genes in replicated microarray experiments.. Bioinformatics.

[44] Wagner A (2000). Inferring lifestyle from gene expression patterns.. Mol Biol Evol.

[45] Bennetzen JL, Hall BD (1982). Codon selection in yeast.. J Biol Chem.

[46] Chan PP, Lowe TM (2009). GtRNAdb: a database of transfer RNA genes detected in genomic sequence.. Nucleic Acids Res.

[47] Rozenski J, Crain PF, McCloskey JA (1999). The RNA Modification Database: 1999 update.. Nucleic Acids Res.

[48] Agris PF (2004). Decoding the genome: a modified view.. Nucleic Acids Res.

[49] Agris PF, Vendeix FA, Graham WD (2007). tRNA's wobble decoding of the genome: 40 years of modification.. J Mol Biol.

[50] Meier F, Suter B, Grosjean H, Keith G, Kubli E (1985). Queuosine modification of the wobble base in tRNAHis influences ‘in vivo’ decoding properties.. EMBO J.

[51] Kruger MK, Pedersen S, Hagervall TG, Sorensen MA (1998). The modification of the wobble base of tRNAGlu modulates the translation rate of glutamic acid codons in vivo.. J Mol Biol.

[52] Grosjean H, de Crecy-Lagard V, Marck C (2009). Deciphering synonymous codons in the three domains of life: Co-evolution with specific tRNA modification enzymes.. FEBS Lett.

[53] Hershberg R, Petrov DA (2009). General rules for optimal codon choice.. PLoS Genet.

[54] Koonin EV, Wolf YI (2008). Genomics of bacteria and archaea: the emerging dynamic view of the prokaryotic world.. Nucleic Acids Res.

[55] Ranea JA, Grant A, Thornton JM, Orengo CA (2005). Microeconomic principles explain an optimal genome size in bacteria.. Trends Genet.

[56] Rocha EP (2004). Codon usage bias from tRNA's point of view: redundancy, specialization, and efficient decoding for translation optimization.. Genome Res.

[57] Karlin S, Mrazek J, Ma J, Brocchieri L (2005). Predicted highly expressed genes in archaeal genomes.. Proc Natl Acad Sci U S A.

[58] Kanaya S, Yamada Y, Kinouchi M, Kudo Y, Ikemura T (2001). Codon usage and tRNA genes in eukaryotes: correlation of codon usage diversity with translation efficiency and with CG-dinucleotide usage as assessed by multivariate analysis.. J Mol Evol.

[59] Ishihama Y, Schmidt T, Rappsilber J, Mann M, Hartl FU (2008). Protein abundance profiling of the Escherichia coli cytosol.. BMC Genomics.

[60] Roy H, Becker HD, Reinbolt J, Kern D (2003). When contemporary aminoacyl-tRNA synthetases invent their cognate amino acid metabolism.. Proc Natl Acad Sci U S A.

[61] Hess DC, Lu W, Rabinowitz JD, Botstein D (2006). Ammonium toxicity and potassium limitation in yeast.. PLoS Biol.

[62] Seaver LC, Imlay JA (2001). Alkyl hydroperoxide reductase is the primary scavenger of endogenous hydrogen peroxide in Escherichia coli.. J Bacteriol.

[63] Glyakina AV, Garbuzynskiy SO, Lobanov MY, Galzitskaya OV (2007). Different packing of external residues can explain differences in the thermostability of proteins from thermophilic and mesophilic organisms.. Bioinformatics.

[64] Mizuguchi K, Sele M, Cubellis MV (2007). Environment specific substitution tables for thermophilic proteins.. BMC Bioinformatics.

[65] D'Amico S, Collins T, Marx JC, Feller G, Gerday C (2006). Psychrophilic microorganisms: challenges for life.. EMBO Rep.

[66] ftp://ftp.ncbi.nih.gov/genomes/Bacteria/

[67] Selengut JD, Haft DH, Davidsen T, Ganapathy A, Gwinn-Giglio M (2007). TIGRFAMs and Genome Properties: tools for the assignment of molecular function and biological process in prokaryotic genomes.. Nucleic Acids Res.

[68] http://www.ncbi.nlm.nih.gov/genomes/lproks.cgi

[69] Fawcett T (2006). An introduction to ROC analysis.. Pattern Recognition Letters.

[70] http://fast-random-forest.googlecode.com/

[71] Lu P, Vogel C, Wang R, Yao X, Marcotte EM (2007). Absolute protein expression profiling estimates the relative contributions of transcriptional and translational regulation.. Nat Biotechnol.

[72] McDonald JH (2008). Sign test. Handbook of Biological Statistics..

[73] Nadeau C, Bengio Y (2003). Inference for the generalization error.. Machine Learning.

[74] Chen K, Roberts E, Luthey-Schulten Z (2009). Horizontal gene transfer of zinc and non-zinc forms of bacterial ribosomal protein S4.. BMC Evol Biol.

[75] Molina N, van Nimwegen E (2008). Universal patterns of purifying selection at noncoding positions in bacteria.. Genome Res.

[76] Langille MG, Brinkman FS (2009). IslandViewer: an integrated interface for computational identification and visualization of genomic islands.. Bioinformatics.

[77] McDonald JH (2008). Fisher's exact test of independence. Handbook of Biological Statistics..

[78] Ashburner M, Ball CA, Blake JA, Botstein D, Butler H (2000). Gene ontology: tool for the unification of biology. The Gene Ontology Consortium.. Nat Genet.

[79] Tatusov RL, Fedorova ND, Jackson JD, Jacobs AR, Kiryutin B (2003). The COG database: an updated version includes eukaryotes.. BMC Bioinformatics.

