# Supplementary figures and images for: Translational Selection Is Ubiquitous in Prokaryotes
Source: PLoS Genet. 2010 Jun 24;6(6):e1001004. doi: 10.1371/journal.pgen.1001004 (PMC2891978; doi:10.1371/journal.pgen.1001004)

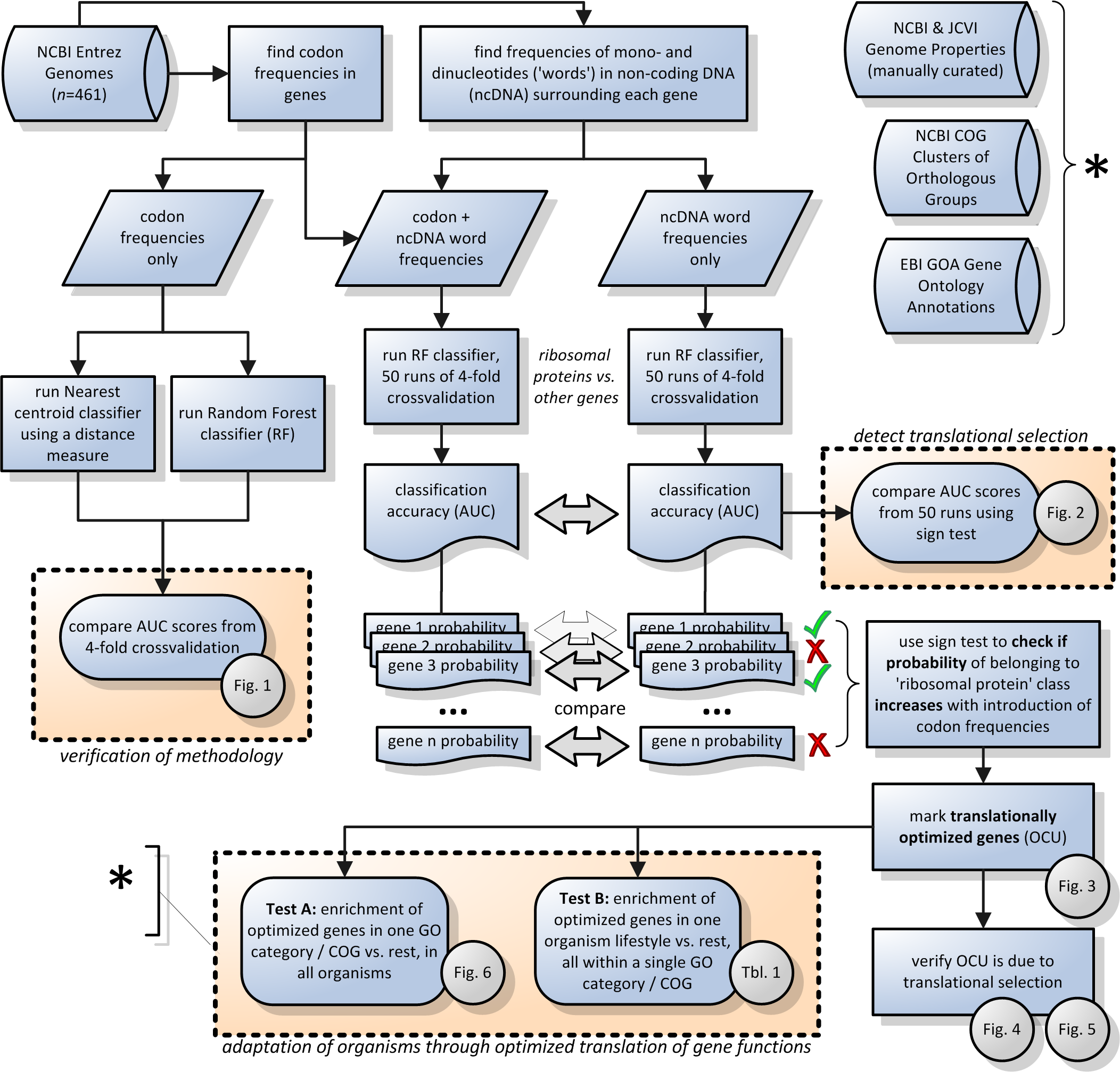

Supplement: Figure S1 — The workflow of the computational framework for detecting translational selection. Cylinders represent databases, rectangles represent operations and/or computation, parallelograms represent datasets, rounded rectangles within dotted frames represent endpoints, circles are references to Figures and Tables. (2.72 MB TIF) [file pgen.1001004.s003.tif]

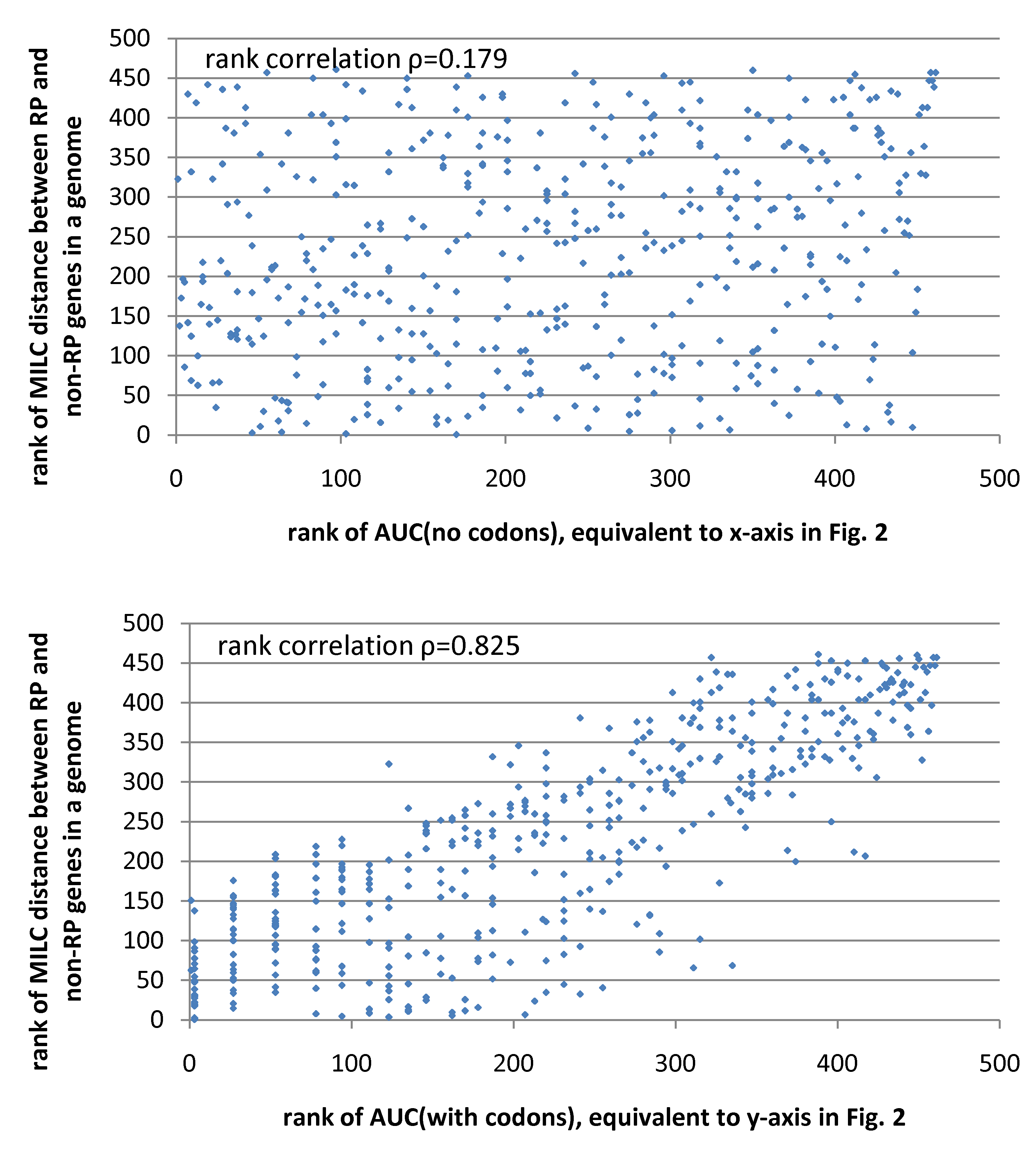

Supplement: Figure S3 — Correlation of the crossvalidation accuracy of RF classifiers with an estimate of intensity of genome-wide codon biases. Accuracy (as AUC score) of RF models trained on composition of intergenic DNA (top) and on composition of intergenic DNA plus codon frequencies (below) is compared to the MILC measure of distance between codon frequencies of ribosomal protein genes, and the rest of the genes within a genome. (0.82 MB TIF) [file pgen.1001004.s005.tif]
