# Supplementary material for: Translational Selection Is Ubiquitous in Prokaryotes
Source: PLoS Genet. 2010 Jun 24;6(6):e1001004. doi: 10.1371/journal.pgen.1001004 (PMC2891978; doi:10.1371/journal.pgen.1001004)
Supplement: Table S1 — Correlations of E. coli cytoplasmic protein abundances with per-gene class probabilities of Random Forest and three codon distance measures. “Overlapping data” implies that only the genes present in both studies were considered. The Pearson correlation was computed after removing two proteins with extremely high abundance values in the Ishihama dataset. (0.04 MB DOC) [file pgen.1001004.s007.doc]

**Supporting Table S1.** Correlations of *E. coli* cytoplasmic protein abundances with per-gene class probabilities of Random Forest and three codon distance measures. "Overlapping data" implies that only the genes present in both studies were considered. The Pearson correlation (*) was computed after removing two proteins with extremely high abundance values in the Ishihama dataset.

| Overlapping data, Spearman | | | | |
| --- | --- | --- | --- | --- |
|  | CB | CAI | MILC | RF |
| Ishihama (*n*=369) | 0.74 | 0.7 | 0.75 | 0.77 |
| Lu (*n*=369) | 0.56 | 0.53 | 0.56 | 0.61 |
| Full data, Spearman | | | | |
|  | CB | CAI | MILC | RF |
| Ishihama (*n*=1047) | 0.68 | 0.63 | 0.64 | 0.64 |
| Lu (*n*=436) | 0.58 | 0.55 | 0.58 | 0.60 |
| Full data passing quality check for Ishihama (ion count >= 4), Spearman | | | | |
|  | CB | CAI | MILC | RF |
| Ishihama (*n*=725) | 0.67 | 0.61 | 0.64 | 0.69 |
|  | | | | |
| Overlapping data, Pearson * | | | | |
|  | CB | CAI | MILC | RF |
| Ishihama (*n*=367) | 0.29 | 0.25 | 0.33 | 0.39 |
| Lu (*n*=367) | 0.42 | 0.39 | 0.45 | 0.47 |
| Full data, Pearson * | | | | |
|  | CB | CAI | MILC | RF |
| Ishihama (*n*=1045) | 0.28 | 0.24 | 0.32 | 0.40 |
| Lu (*n*=434) | 0.44 | 0.40 | 0.47 | 0.49 |
| Full data passing quality check for Ishihama (ion count >= 4), Pearson * | | | | |
|  | CB | CAI | MILC | RF |
| Ishihama (*n*=723) | 0.28 | 0.24 | 0.32 | 0.39 |

**References:**

[1] Ishihama Y, Schmidt T, Rappsilber J, Mann M, Hartl FU, et al. (2008) Protein abundance profiling of the Escherichia coli cytosol. BMC Genomics 9: 102.

[2] Lu P, Vogel C, Wang R, Yao X, Marcotte EM (2007) Absolute protein expression profiling estimates the relative contributions of transcriptional and translational regulation. Nat Biotechnol 25: 117-124.
