# Supplementary material for: Translational Selection Is Ubiquitous in Prokaryotes
Source: PLoS Genet. 2010 Jun 24;6(6):e1001004. doi: 10.1371/journal.pgen.1001004 (PMC2891978; doi:10.1371/journal.pgen.1001004)
Supplement: Table S2 — A survey of the amount of non-coding DNA within bacterial and archaeal genomes. Alongside the data for ten representative genomes, two additional rows display: the median values for the entire set of 461 genomes, and the 1st percentile of values for the 461 genomes. Table cells show the number of non-coding nucleotides in a window size of 10 kilobases upstream of a gene's start codon, and 10 kilobases downstream of the stop codon. (0.04 MB DOC) [file pgen.1001004.s008.doc]

**Supporting Table S2.** A survey of the amount of non-coding DNA within bacterial and archaeal genomes. Alongside the data for ten representative genomes, two additional rows display: the median values for the entire set of 461 genomes, and the 1^st^ percentile of values for the 461 genomes. Table cells show the number of non-coding nucleotides in a window size of 10 kilobases upstream of a gene’s start codon, and 10 kilobases downstream of the stop codon.

| organism | minimum | 1st perc. | 2nd perc. | 5th perc. | 10th perc. | 25th perc. | median |
| --- | --- | --- | --- | --- | --- | --- | --- |
| *Bacillus subtilis* | 15 | 881 | 1018 | 1298 | 1489 | 1864 | 2283 |
| *Borrelia burgdorferi* | 226 | 286 | 351 | 494 | 683 | 964 | 1490 |
| *Deinococcus radiodurans* | 363 | 790 | 912 | 1055 | 1231 | 1542 | 1969 |
| *Escherichia coli K12* | 523 | 1089 | 1160 | 1381 | 1568 | 1983 | 2450 |
| *Halobacterium sp.* | 514 | 869 | 937 | 1119 | 1292 | 1629 | 2183 |
| *Helicobacter pylori 26695* | 216 | 346 | 416 | 718 | 935 | 1277 | 1817 |
| *Mycoplasma genitalium* | 151 | 173 | 175 | 282 | 332 | 558 | 841 |
| *Pelagibacter ubique HTCC1062* | 11 | 141 | 192 | 280 | 371 | 510 | 757 |
| *Pseudomonas aeruginosa* | 181 | 820 | 932 | 1127 | 1316 | 1656 | 2107 |
| *Streptomyces coelicolor* | 89 | 826 | 950 | 1168 | 1348 | 1722 | 2194 |
| median of 461 organisms | 355 | 827 | 971 | 1233 | 1443 | 1836 | 2366 |
| 1^st^ percentile of 461 organisms | 3.2 | 142.2 | 181.0 | 281.2 | 364.6 | 554.0 | 848.2 |
