# Supplementary material for: Translational Selection Is Ubiquitous in Prokaryotes
Source: PLoS Genet. 2010 Jun 24;6(6):e1001004. doi: 10.1371/journal.pgen.1001004 (PMC2891978; doi:10.1371/journal.pgen.1001004)
Supplement: Table S3 — Accuracy of the RF classifier in the task of discriminating ribosomal protein genes. Accuracy is expressed as area-under-ROC-curve (AUC) score, and given for RF classifiers trained without codon frequencies (“AUC, no codons”) and with codon frequencies (“AUC, with codons”). Mean and standard deviation of AUC are computed from 50 runs of crossvalidation. The sign test p-value indicates whether the AUC score exhibits a statistically significant increase with introduction of codon frequencies to the classifier. Ten representative genomes are shown, along with three genomes with the least significant p-values among all 461 genomes. Full data is available as Dataset S1, or from the website http://www.adaptome.org/. (0.05 MB DOC) [file pgen.1001004.s009.doc]

**Supporting Table S3.** Accuracy of the RF classifier in the task of discriminating ribosomal protein genes. Accuracy is expressed as area-under-ROC-curve (AUC) score, and given for RF classifiers trained without codon frequencies (“AUC, no codons”) and with codon frequencies (“AUC, with codons”). Mean and standard deviation of AUC are computed from 50 runs of crossvalidation. The sign test *p*-value indicates whether the AUC score exhibits a statistically significant increase with introduction of codon frequencies to the classifier. Ten representative genomes are shown, along with three genomes with the least significant *p*-values among all 461 genomes. Full data is available as Supporting Dataset S1, or from the website <http://www.adaptome.org/>.

|  | gene count * | AUC, no codons  (mean ± st. dev.) | AUC, with codons  (mean ± st. dev.) | sign test  *p*-value |
| --- | --- | --- | --- | --- |
| *Bacillus subtilis* | 3814 | 0.847 ± 0.021 | 0.977 ± 0.004 | 8.88 x 10^-16^ |
| *Borrelia burgdorferi* | 1384 | 0.896 ± 0.014 | 0.948 ± 0.006 | 8.88 x 10^-16^ |
| *Deinococcus radiodurans* | 3096 | 0.873 ± 0.021 | 0.987 ± 0.002 | 8.88 x 10^-16^ |
| *Escherichia coli K12* | 3914 | 0.877 ± 0.027 | 0.997 ± <0.001 | 8.88 x 10^-16^ |
| *Halobacterium sp.* | 2391 | 0.866 ± 0.015 | 0.954 ± 0.008 | 8.88 x 10^-16^ |
| *Helicobacter pylori 26695* | 1470 | 0.794 ± 0.022 | 0.900 ± 0.007 | 8.88 x 10^-16^ |
| *Mycoplasma genitalium* | 465 | 0.823 ± 0.020 | 0.938 ± 0.006 | 8.88 x 10^-16^ |
| *Pelagibacter ubique HTCC1062* | 1292 | 0.808 ± 0.016 | 0.939 ± 0.008 | 8.88 x 10^-16^ |
| *Pseudomonas aeruginosa* | 5426 | 0.926 ± 0.015 | 0.997 ± 0.001 | 8.88 x 10^-16^ |
| *Streptomyces coelicolor* | 7772 | 0.801 ± 0.016 | 0.986 ± 0.003 | 8.88 x 10^-16^ |
| **Three least significant results:** *Salinibacter ruber DSM 13855* | 2745 | 0.922 ± 0.017 | 0.942 ± 0.008 | 1.62 x 10^-8^ |
| *Rickettsia rickettsii Sheila Smith* | 1010 | 0.885 ± 0.020 | 0.900 ± 0.008 | 1.19 x 10^-5^ |
| *Saccharophagus degradans 2-40* | 3929 | 0.822 ± 0.014 | 0.822 ± 0.023 | 8.39 x 10^-1^ |
| minimum ** | 161 | 0.467 | 0.768 | 8.88 x 10^-16^ |
| median ** | 2854 | 0.854 | 0.978 | 8.88 x 10^-16^ |
| maximum ** | 9178 | 0.946 | 0.999 | 8.39 x 10^-1^ |

* Genes shorter than 80 codons are excluded from all computations, and therefore also from this count.

** The “minimum”, “median” and “maximum” are given for each column separately; therefore, in these rows, one row does not represent a single organism.
