# Supplementary material for: Translational Selection Is Ubiquitous in Prokaryotes
Source: PLoS Genet. 2010 Jun 24;6(6):e1001004. doi: 10.1371/journal.pgen.1001004 (PMC2891978; doi:10.1371/journal.pgen.1001004)
Supplement: Table S4 — Microarray signal intensities for ribosomal protein genes and aminoacyl-tRNA synthetases (aa-tRS). See Text S1, Appendix 1 for detailed information of which NCBI GEO Samples were chosen from the NCBI GEO Series in the table. (0.05 MB DOC) [file pgen.1001004.s010.doc]

**Supporting Table S4.** Microarray signal intensities for ribosomal protein genes and aminoacyl-tRNA synthetases (aa-tRS). See Supporting Text S1, Appendix 1 for detailed information of which NCBI GEO Samples were chosen from the NCBI GEO Series in the table.

| NCBI GEO Series Id | organism name | average expression | ribosomal protein avg. signal | ratio ribo/avg | aa‑tRs avg. signal | ratio aa‑tRS/avg |
| --- | --- | --- | --- | --- | --- | --- |
| GSE4026 | *Pseudomonas aeruginosa* | 3337.59 | 24792.57 | 7.43 x | 4399.99 | 1.32 x |
| GSE2728 | *Staphylococcus aureus* Mu50 | 5698.29 | 13139.89 | 2.31 x | 3356.31 | 0.59 x |
| GSE7588 | *Mycobacterium tuberculosis* H37Rv | 649.88 | 2802.48 | 4.31 x | 414.52 | 0.64 x |
| GSE5865 | *Bifidobacterium longum* | 15460.44 | 100434.45 | 6.50 x | 9641.74 | 0.62 x |
| GSE12491 | *Bradyrhizobium japonicum* | 688.01 | 1777.57 | 2.58 x | 586.67 | 0.85 x |
| GSE5061 | *Haemophilus influenza* | 5544.94 | 24819.15 | 4.48 x | 7705.99 | 1.39 x |
| GSE11383 | *Lactobacillus plantarum* | 1001.78 | 3227.09 | 3.22 x | 818.02 | 0.82 x |
| GSE3247 | *Listeria monocytogenes* | 2.52 | 12.29 | 4.87 x | 3.57 | 1.42 x |
| GSE10507 | *Nitrosomonas europaea* | 685.23 | 3689.94 | 5.38 x | 595.94 | 0.87 x |
| GSE4848 | *Pseudomonas syringae tomato* DC3000 | 1001.92 | 4561.96 | 4.55 x | 1457.12 | 1.45 x |
| GSE12269 | *Rhodobacter sphaeroides* 2.4.1 | 375.50 | 4540.38 | 12.09 x | 467.36 | 1.24 x |
| GSE6221 | *Rhodopseudomonas palustris* CGA009 | 786.04 | 2799.51 | 3.56 x | 622.01 | 0.79 x |
| GSE6973 | *Streptococcus mutans* | 981.62 | 1901.71 | 1.94 x | 1446.93 | 1.47 x |
| GSE10368 | *Thermus thermophilus* HB8 | 797.42 | 5331.28 | 6.69 x | 739.80 | 0.93 x |
| GSE11937 | *Bacillus subtilis* | 1.00 | 9.92 | 9.95 x | 1.29 | 1.29 x |
| GSE13982 | *Escherichia coli* K12 | 1515.13 | 10325.07 | 6.81 x | 2584.16 | 1.71 x |
| GSE7172 | *Streptomyces coelicolor* | 1.08 | 16.35 | 15.17 x | 1.16 | 1.08 x |
| GSE4447 | *Desulfovibrio vulgaris* Hildenborough | 1.15 | 5.42 | 4.72 x | 0.80 | 0.69 x |
| GSE4631 | *Salmonella typhimurium* LT2 | 0.24 | 1.85 | 7.71 x | 0.38 | 1.60 x |
|  |  |  | on average | ***6.01 x*** | on average | ***1.09 x*** |
