# Supplementary material for: Translational Selection Is Ubiquitous in Prokaryotes
Source: PLoS Genet. 2010 Jun 24;6(6):e1001004. doi: 10.1371/journal.pgen.1001004 (PMC2891978; doi:10.1371/journal.pgen.1001004)
Supplement: Table S5 — Differences in microarray signal intensities for OCU and non-OCU genes. “BWS” stands for Baumgartner-Weiss-Schindler permutation test used to determine if the distribution of microarray signal intensities of OCU genes is shifted in comparison to the distribution of microarray signal intensities of non-OCU genes. (0.05 MB DOC) [file pgen.1001004.s011.doc]

**Supporting Table S5.** Differences in microarray signal intensities for OCU and non-OCU genes. BWS stands for Baumgartner-Weiss-Schindler permutation test [1] used to determine if the distribution of microarray signal intensities of OCU genes is shifted in comparison to the distribution of microarray signal intensities of non-OCU genes.

| NCBI GEO Series Id | organism name | BWS [1]  *p*-value | OCU average signal | non-OCU  average signal | ratio  OCU /  non‑OCU | ratio OCU /non-OCU, ribo. prot. excluded |
| --- | --- | --- | --- | --- | --- | --- |
| GSE4026 | *Pseudomonas aeruginosa ** | < 1·10^-6^ | 10656.48 | 2856.97 | 3.73 x | 3.57 x |
| GSE2728 | *Staphylococcus aureus* Mu50 | < 1·10^-6^ | 13757.60 | 4789.06 | 2.87 x | 2.84 x |
| GSE7588 | *Mycobacterium tuberculosis* H37Rv * | 3.0·10^-3^ | 840.29 | 611.08 | 1.38 x | 1.38 x |
| GSE5865 | *Bifidobacterium longum* | < 1·10^-6^ | 37573.21 | 12092.17 | 3.11 x | 3.23 x |
| GSE12491 | *Bradyrhizobium japonicum* | < 1·10^-6^ | 1333.26 | 623.07 | 2.14 x | 2.12 x |
| GSE5061 | *Haemophilus influenzae* | < 1·10^-6^ | 11538.77 | 4875.66 | 2.37 x | 2.10 x |
| GSE11383 | *Lactobacillus plantarum* | 2.0·10^-6^ | 1586.71 | 942.90 | 1.68 x | 1.61 x |
| GSE3247 | *Listeria monocytogenes* | < 1·10^-6^ | 5.74 | 2.09 | 2.74 x | 2.60 x |
| GSE10507 | *Nitrosomonas europaea* * | < 1·10^-6^ | 821.12 | 660.29 | 1.24 x | 1.56 x |
| GSE4848 | *Pseudomonas syringae tomato* DC3000 | 1.1·10^-3^ | 1296.65 | 975.04 | 1.33 x | 1.27 x |
| GSE12269 | *Rhodobacter sphaeroides* 2.4.1 | < 1·10^-6^ | 882.83 | 310.67 | 2.84 x | 2.69 x |
| GSE6221 | *Rhodopseudomonas palustris* CGA009 | < 1·10^-6^ | 1415.86 | 697.83 | 2.03 x | 2.07 x |
| GSE6973 | *Streptococcus mutans* | < 1·10^-6^ | 1346.96 | 950.66 | 1.42 x | 1.31 x |
| GSE10368 | *Thermus thermophilus* HB8 | < 1·10^-6^ | 1444.96 | 702.88 | 2.06 x | 1.76 x |
| GSE11937 | *Bacillus subtilis* | 2.4·10^-5^ | 1.65 | 0.94 | 1.76 x | 1.41 x |
| GSE13982 | *Escherichia coli* K12 | < 1·10^-6^ | 10325.07 | 1515.13 | 3.18 x | 3.16 x |
| GSE7172 | *Streptomyces coelicolor* * | < 1·10^-6^ | 2.97 | 0.95 | 3.13 x | 2.53 x |
| GSE4447 | *Desulfovibrio vulgaris* Hildenborough | < 1·10^-6^ | 2.25 | 0.98 | 2.31 x | 2.14 x |
| GSE4631 | *Salmonella typhimurium* LT2 | < 1·10^-6^ | 0.68 | 0.21 | 3.33 x | 3.22 x |
|  | median *p*-value | ***< 1·10^-6^*** | avg. ratio | | ***2.36 x*** | ***2.24 x*** |

* In these organisms, translational selection was not detected in at least 2 out of 3 previous multi-genome studies; see Supplementary Appendix B.

**Reference:**

[1] Neuhauser M, Senske R (2004) The Baumgartner-Weiss-Schindler test for the detection of differentially expressed genes in replicated microarray experiments. Bioinformatics 20: 3553-3564.
