# Supplementary material for: Translational Selection Is Ubiquitous in Prokaryotes
Source: PLoS Genet. 2010 Jun 24;6(6):e1001004. doi: 10.1371/journal.pgen.1001004 (PMC2891978; doi:10.1371/journal.pgen.1001004)
Supplement: Table S6 — Preferences of OCU genes towards optimal or sub-optimal codons for two-fold amino acids, as defined by the genome's tRNA gene content. Table cells show the number of genomes where OCU genes prefer the optimal codon, the suboptimal codon, or where there is no preference towards either codon. Preference for codons is detected by the Mann-Whitney U test on codon frequencies of OCU versus non-OCU genes, at p<10−3. Optimal codons are those directly recognized by the anticodon of a tRNA encoded in the genome [1], and the suboptimal codons, conversely, have no tRNA with the appropriate anticodon. In some cases (frequently for Lys, Gln and Glu), genomes may encode tRNAs with both anticodons and the optimal/suboptimal anticodon cannot be defined; therefore the “sum” column may be lesser than the total number of genomes. (0.04 MB DOC) [file pgen.1001004.s012.doc]

**Supporting Table S6.** Preferences of OCU genes towards optimal or sub-optimal codons for two-fold amino acids, as defined by the genome's tRNA gene content. Table cells show the number of genomes where OCU genes prefer the optimal codon, the suboptimal codon, or where there is no preference towards either codon. Preference for codons is detected by the Mann-Whitney U test on codon frequencies of OCU vs. non-OCU genes, at *p*<10^-3^. Optimal codons are those directly recognized by the anticodon of a tRNA encoded in the genome [1], and the suboptimal codons, conversely, have no tRNA with the appropriate anticodon. In some cases (frequently for Lys, Gln and Glu), genomes may encode tRNAs with both anticodons and the optimal/suboptimal anticodon cannot be defined; therefore the "sum" column may be lesser than the total number of genomes.

|  | **Bacteria (*n* = 415)** | | | | **Archaea (*n* = 46*)** | | | |
| --- | --- | --- | --- | --- | --- | --- | --- | --- |
| a.a. | optimal codon | diff. not signif. | subopt. codon | sum | optimal codon | diff. not signif. | subopt. codon | sum |
| *Phe* | 234 | 149 | 32 | 415 | 30 | 7 | 8 | 45 |
| *Tyr* | 240 | 150 | 23 | 413 | 26 | 11 | 8 | 45 |
| *Cys* | 46 | 314 | 55 | 415 | 0 | 40 | 5 | 45 |
| *His* | 207 | 185 | 22 | 414 | 23 | 16 | 6 | 45 |
| *Gln* | 146 | 76 | 13 | 235 | 2 | 3 | 1 | 6 |
| *Asn* | 251 | 141 | 23 | 415 | 25 | 12 | 8 | 45 |
| *Lys* | 60 | 90 | 17 | 167 | 3 | 4 | 2 | 9 |
| *Asp* | 188 | 176 | 51 | 415 | 26 | 13 | 6 | 45 |
| *Glu* | 177 | 76 | 13 | 266 | 5 | 2 | 1 | 8 |
| all | 1549 | 1357 | 249 | 3155 | 140 | 108 | 45 | 293 |
| optimal-to-suboptimal ratio = 6.22 x  ratio *p* < 10^-30^ (by sign test) | | | |  | optimal-to-suboptimal ratio = 3.11 x  ratio *p* = 7.6·10^-13^ (by sign test) | | |  |

* The archaeon *Caldivirga maquilingensis* IC-167 could not be matched to a record in the Genomic tRNA Database [1] at the time of writing; therefore, this number is effectively 45.

**Reference:**

[1] Chan PP, Lowe TM (2009) GtRNAdb: a database of transfer RNA genes detected in genomic sequence. Nucleic Acids Res 37: D93-97. <http://gtrnadb.ucsc.edu/>
