# Supplementary material for: Translational Selection Is Ubiquitous in Prokaryotes
Source: PLoS Genet. 2010 Jun 24;6(6):e1001004. doi: 10.1371/journal.pgen.1001004 (PMC2891978; doi:10.1371/journal.pgen.1001004)
Supplement: Table S7 — Protein functional categories enriched with (or depleted of) OCU genes in the “molecular function” namespace of the Gene Ontology. The list is filtered to exclude a number of categories redundant to the ones displayed using the REViGO tool available at http://revigo.irb.hr/; for a complete listing, please refer to Dataset S2, or the authors' website at http://www.adaptome.org (0.16 MB DOC) [file pgen.1001004.s013.doc]

**Supporting** **Table S7.** Protein functional categories enriched with (or depleted of) OCU genes in the “molecular function” namespace of the Gene Ontology. The list is filtered to exclude a number of categories redundant to the ones displayed using the REViGO tool available at <http://revigo.irb.hr/> ; for a complete listing, please refer to Supporting Dataset S2, or the authors’ website at [http://www.adaptome.org](http://www.adaptome.org/)

| ***Bacteria*** | | | |  | | |  | | |  | |  | |  | | | |
| --- | --- | --- | --- | --- | --- | --- | --- | --- | --- | --- | --- | --- | --- | --- | --- | --- | --- |
| Group ID | | | n(OCU) | | | n(others) | | | log(pVal) | | enrich | | Description | | | |  |
|  | | GO:0004326 | 0 | | 535 | | | -27.0 | | | 0.00 | | tetrahydrofolylpolyglutamate synthase activity | | | | |
|  | | GO:0004356 | 197 | | 664 | | | -22.0 | | | 2.07 | | glutamate-ammonia ligase activity | | | | |
|  | | GO:0004775 | 288 | | 376 | | | -98.5 | | | 3.92 | | succinate-CoA ligase (ADP-forming) activity | | | | |
|  | | GO:0004817 | 1 | | 473 | | | -22.2 | | | 0.02 | | cysteine-tRNA ligase activity | | | | |
|  | | GO:0015035 | 1002 | | 1652 | | | -280.0 | | | 3.42 | | protein disulfide oxidoreductase activity | | | | |
|  | | GO:0004412 | 1 | | 438 | | | -20.4 | | | 0.02 | | homoserine dehydrogenase activity | | | | |
|  | | GO:0003911 | 0 | | 438 | | | -22.1 | | | 0.00 | | DNA ligase (NAD+) activity | | | | |
|  | | GO:0000155 | 39 | | 14283 | | | -300.0 | | | 0.02 | | two-component sensor activity | | | | |
|  | | GO:0004455 | 172 | | 177 | | | -69.6 | | | 4.45 | | ketol-acid reductoisomerase activity | | | | |
|  | | GO:0004365 | 245 | | 443 | | | -63.5 | | | 3.22 | | glyceraldehyde-3-phosphate dehydrogenase (phosphorylating) activity | | | | |
|  | | GO:0004784 | 300 | | 336 | | | -114.2 | | | 4.26 | | superoxide dismutase activity | | | | |
|  | | GO:0000104 | 249 | | 570 | | | -49.9 | | | 2.75 | | succinate dehydrogenase activity | | | | |
|  | | GO:0051920 | 151 | | 198 | | | -52.0 | | | 3.91 | | peroxiredoxin activity | | | | |
|  | | GO:0004149 | 97 | | 238 | | | -18.4 | | | 2.61 | | dihydrolipoyllysine-residue succinyltransferase activity | | | | |
|  | | GO:0016679 | 205 | | 558 | | | -32.8 | | | 2.43 | | oxidoreductase activity acting on diphenols and related substances as donors | | | | |
|  | | GO:0004634 | 177 | | 248 | | | -57.7 | | | 3.76 | | phosphopyruvate hydratase activity | | | | |
|  | | GO:0003840 | 1 | | 446 | | | -20.7 | | | 0.02 | | gamma-glutamyltransferase activity | | | | |
|  | | GO:0004332 | 169 | | 411 | | | -31.7 | | | 2.63 | | fructose-bisphosphate aldolase activity | | | | |
|  | | GO:0004618 | 115 | | 297 | | | -20.2 | | | 2.52 | | phosphoglycerate kinase activity | | | | |
|  | | GO:0030611 | 83 | | 211 | | | -15.2 | | | 2.55 | | arsenate reductase activity | | | | |
|  | | GO:0004550 | 249 | | 126 | | | -141.7 | | | 6.00 | | nucleoside diphosphate kinase activity | | | | |
|  | | GO:0004746 | 162 | | 376 | | | -32.3 | | | 2.72 | | riboflavin synthase activity | | | | |
|  | | GO:0004160 | 1 | | 473 | | | -22.2 | | | 0.02 | | dihydroxy-acid dehydratase activity | | | | |
|  | | GO:0003878 | 169 | | 176 | | | -67.9 | | | 4.42 | | ATP citrate synthase activity | | | | |
|  | | GO:0008955 | 0 | | 549 | | | -27.7 | | | 0.00 | | peptidoglycan glycosyltransferase activity | | | | |
|  | | GO:0003866 | 0 | | 383 | | | -19.2 | | | 0.00 | | 3-phosphoshikimate 1-carboxyvinyltransferase activity | | | | |
|  | | GO:0004633 | 1 | | 357 | | | -16.4 | | | 0.03 | | phosphopantothenoylcysteine decarboxylase activity | | | | |
|  | | GO:0004794 | 2 | | 408 | | | -17.6 | | | 0.04 | | L-threonine ammonia-lyase activity | | | | |
|  | | GO:0019134 | 0 | | 322 | | | -16.2 | | | 0.00 | | glucosamine-1-phosphate N-acetyltransferase activity | | | | |
|  | | GO:0004462 | 122 | | 295 | | | -23.4 | | | 2.64 | | lactoylglutathione lyase activity | | | | |
|  | | GO:0003977 | 1 | | 355 | | | -16.2 | | | 0.03 | | UDP-N-acetylglucosamine diphosphorylase activity | | | | |
|  | | GO:0008898 | 0 | | 416 | | | -21.0 | | | 0.00 | | homocysteine S-methyltransferase activity | | | | |
|  | | GO:0004479 | 2 | | 424 | | | -18.3 | | | 0.04 | | methionyl-tRNA formyltransferase activity | | | | |
|  | | GO:0003755 | 864 | | 1789 | | | -191.7 | | | 2.95 | | peptidyl-prolyl cis-trans isomerase activity | | | | |
|  | | GO:0008784 | 2 | | 580 | | | -26.1 | | | 0.03 | | alanine racemase activity | | | | |
|  | | GO:0004493 | 38 | | 43 | | | -15.2 | | | 4.23 | | methylmalonyl-CoA epimerase activity | | | | |
|  | | GO:0046538 | 61 | | 117 | | | -15.6 | | | 3.09 | | 2,3-bisphosphoglycerate-dependent phosphoglycerate mutase activity | | | | |
|  | | GO:0004133 | 3 | | 563 | | | -23.8 | | | 0.05 | | glycogen debranching enzyme activity | | | | |
|  | | GO:0009381 | 0 | | 1285 | | | -65.3 | | | 0.00 | | excinuclease ABC activity | | | | |
|  | | GO:0008409 | 2 | | 883 | | | -41.1 | | | 0.02 | | 5'-3' exonuclease activity | | | | |
|  | | GO:0008484 | 2 | | 1170 | | | -55.4 | | | 0.02 | | sulfuric ester hydrolase activity | | | | |
|  | | GO:0004526 | 105 | | 278 | | | -18.0 | | | 2.47 | | ribonuclease P activity | | | | |
|  | | GO:0004563 | 0 | | 328 | | | -16.6 | | | 0.00 | | beta-N-acetylhexosaminidase activity | | | | |
|  | | GO:0009039 | 122 | | 359 | | | -17.7 | | | 2.29 | | urease activity | | | | |
|  | | GO:0030234 | 416 | | 1463 | | | -42.0 | | | 2.00 | | enzyme regulator activity | | | | |
|  | | GO:0045156 | 125 | | 61 | | | -72.6 | | | 6.07 | | electron transporter, transferring electrons within the cyclic electron transport pathway of photosynthesis activity | | | | |
|  | | GO:0045158 | 39 | | 12 | | | -26.7 | | | 6.90 | | electron transporter, transferring electrons within cytochrome b6 f complex of photosystem II activity | | | | |
|  | | GO:0003735 | 6879 | | 10736 | | | -300.0 | | | 3.65 | | structural constituent of ribosome | | | | |
|  | | GO:0016168 | 53 | | 56 | | | -21.8 | | | 4.39 | | chlorophyll binding | | | | |
|  | | GO:0008199 | 321 | | 834 | | | -54.1 | | | 2.51 | | ferric iron binding | | | | |
|  | | GO:0045152 | 142 | | 216 | | | -43.5 | | | 3.58 | | antisigma factor binding | | | | |
|  | | GO:0008134 | 10 | | 2967 | | | -132.6 | | | 0.03 | | transcription factor binding | | | | |
|  | | GO:0051082 | 784 | | 2479 | | | -95.8 | | | 2.17 | | unfolded protein binding | | | | |
|  | | GO:0031405 | 415 | | 812 | | | -98.5 | | | 3.06 | | lipoic acid binding | | | | |
|  | | GO:0030983 | 3 | | 914 | | | -41.1 | | | 0.03 | | mismatched DNA binding | | | | |
|  | | GO:0003697 | 324 | | 1102 | | | -35.1 | | | 2.05 | | single-stranded DNA binding | | | | |
|  | | GO:0019843 | 3812 | | 8905 | | | -300.0 | | | 2.75 | | rRNA binding | | | | |
|  | | GO:0003746 | 1109 | | 1687 | | | -300.0 | | | 3.60 | | translation elongation factor activity | | | | |
|  | | GO:0004427 | 216 | | 351 | | | -62.0 | | | 3.44 | | inorganic diphosphatase activity | | | | |
|  | | GO:0004003 | 6 | | 2271 | | | -104.1 | | | 0.02 | | ATP-dependent DNA helicase activity | | | | |
|  | | GO:0051183 | 1 | | 569 | | | -26.9 | | | 0.02 | | vitamin transporter activity | | | | |
|  | | GO:0015238 | 11 | | 3009 | | | -133.2 | | | 0.03 | | drug transporter activity | | | | |
|  | | GO:0015288 | 127 | | 275 | | | -27.7 | | | 2.85 | | porin activity | | | | |
|  | | GO:0015343 | 3 | | 1016 | | | -46.1 | | | 0.03 | | siderophore-iron transmembrane transporter activity | | | | |
|  | | GO:0005244 | 4 | | 700 | | | -29.3 | | | 0.05 | | voltage-gated ion channel activity | | | | |
|  | | GO:0015078 | 2099 | | 5138 | | | -300.0 | | | 2.64 | | hydrogen ion transmembrane transporter activity | | | | |
| ***Archaea*** | | | |  | | |  | | |  | |  | |  | | | |
| Group ID | | | n(OCU) | | n(others) | | | log(pVal) | | | enrich | | GO name | | |  | |
|  | GO:0015078 | | 167 | | 302 | | | -21.4 | | | 2.10 | | hydrogen ion transmembrane transporter activity | |  | | |
|  | GO:0046961 | | 130 | | 185 | | | -23.2 | | | 2.43 | | hydrogen ion transporting ATPase activity rotational mechanism | |  | | |
|  | GO:0046933 | | 112 | | 138 | | | -23.7 | | | 2.63 | | hydrogen ion transporting ATP synthase activity rotational mechanism | |  | | |
|  | GO:0046943 | | 8 | | 335 | | | -17.4 | | | 0.14 | | carboxylic acid transmembrane transporter activity | |  | | |
|  | GO:0008026 | | 2 | | 431 | | | -31.5 | | | 0.03 | | ATP-dependent helicase activity | |  | | |
|  | GO:0004386 | | 16 | | 697 | | | -36.7 | | | 0.13 | | helicase activity | |  | | |
|  | GO:0016811 | | 18 | | 495 | | | -21.0 | | | 0.20 | | hydrolase activity, acting on carbon-nitrogen (but not peptide) bonds in linear amides | |  | | |
|  | GO:0003735 | | 783 | | 1360 | | | -104.8 | | | 2.20 | | structural constituent of ribosome | |  | | |
|  | GO:0005198 | | 823 | | 1411 | | | -112.5 | | | 2.22 | | structural molecule activity | |  | | |
|  | GO:0016758 | | 1 | | 233 | | | -17.0 | | | 0.03 | | transferase activity transferring hexosyl groups | |  | | |
|  | GO:0030269 | | 89 | | 35 | | | -40.2 | | | 4.22 | | tetrahydromethanopterin S-methyltransferase activity | |  | | |
|  | GO:0008172 | | 141 | | 144 | | | -35.5 | | | 2.91 | | S-methyltransferase activity | |  | | |
|  | GO:0050524 | | 64 | | 10 | | | -38.1 | | | 5.08 | | coenzyme-B sulfoethylthiotransferase activity | |  | | |
|  | GO:0016782 | | 113 | | 122 | | | -27.2 | | | 2.83 | | transferase activity transferring sulfur-containing groups | |  | | |
|  | GO:0016773 | | 36 | | 1359 | | | -67.1 | | | 0.15 | | phosphotransferase activity alcohol group as acceptor | |  | | |
|  | GO:0004672 | | 13 | | 859 | | | -51.5 | | | 0.09 | | protein kinase activity | |  | | |
|  | GO:0000155 | | 3 | | 640 | | | -46.7 | | | 0.03 | | two-component sensor activity | |  | | |
|  | GO:0004673 | | 3 | | 640 | | | -46.7 | | | 0.03 | | protein histidine kinase activity | |  | | |
