# Supplementary material for: Translational Selection Is Ubiquitous in Prokaryotes
Source: PLoS Genet. 2010 Jun 24;6(6):e1001004. doi: 10.1371/journal.pgen.1001004 (PMC2891978; doi:10.1371/journal.pgen.1001004)
Supplement: Table S8 — A selection of protein functional categories enriched with (or depleted of) OCU genes in the “biological process” namespace of the Gene Ontology. This table is derived only from 38 organisms whose genomes were claimed to lack translational selection in at least 2 of 3 previous large-scale studies, see Appendix A in Text S1 for listing of the 38 genomes. Data in this table is analogous to the data in Figure 6 of the manuscript in all other aspects except for the restricted choice of genomes in this table. (0.07 MB DOC) [file pgen.1001004.s014.doc]

**Supporting** **Table S8.** A selection of protein functional categories enriched with (or depleted of) OCU genes in the “biological process” namespace of the Gene Ontology. This table is derived only from 38 organisms whose genomes were claimed to lack translational selection in at least 2 of 3 previous large-scale studies [1-3], see Appendix A in Supporting Text S1 for listing of the 38 genomes. Data in this table is analogous to the data in Fig. 6 of the manuscript in all other aspects except for the restricted choice of genomes in this table.

| Group ID | n(OCU) | n(others) | log(pVal) | Enrichment | GeneGroupDescription |
| --- | --- | --- | --- | --- | --- |
| GO:0000027 | 31 | 1 | -26.0 | 7.442 | ribosomal large subunit assembly and maintenance |
| GO:0042026 | 26 | 17 | -12.4 | 4.642 | protein refolding |
| GO:0009061 | 17 | 18 | -6.4 | 3.727 | anaerobic respiration |
| GO:0006879 | 24 | 34 | -7.1 | 3.176 | cellular iron ion homeostasis |
| GO:0015986 | 111 | 163 | -29.1 | 3.125 | ATP synthesis coupled proton transport |
| GO:0015979 | 78 | 138 | -17.2 | 2.779 | photosynthesis |
| GO:0006414 | 64 | 115 | -14.0 | 2.75 | translational elongation |
| GO:0005840 | 552 | 1185 | -92.3 | 2.506 | Ribosome |
| GO:0006413 | 37 | 89 | -5.8 | 2.255 | translational initiation |
| GO:0006801 | 26 | 58 | -4.6 | 2.375 | superoxide metabolic process |
| GO:0006119 | 177 | 461 | -22.2 | 2.143 | oxidative phosphorylation |
| GO:0006457 | 145 | 373 | -18.7 | 2.16 | protein folding |
| GO:0006099 | 63 | 226 | -4.5 | 1.674 | tricarboxylic acid cycle |
| GO:0009072 | 17 | 501 | -13.7 | 0.25 | aromatic amino acid family metabolic process |
| GO:0006813 | 6 | 190 | -5.7 | 0.234 | potassium ion transport |
| GO:0006289 | 4 | 151 | -4.9 | 0.198 | nucleotide-excision repair |
| GO:0015833 | 4 | 162 | -5.6 | 0.184 | peptide transport |
| GO:0043039 | 15 | 810 | -30.8 | 0.138 | tRNA aminoacylation |
| GO:0006865 | 5 | 314 | -12.8 | 0.12 | amino acid transport |
| GO:0050793 | 12 | 524 | -18.2 | 0.171 | regulation of developmental process |
| GO:0006261 | 4 | 283 | -12.0 | 0.107 | DNA-dependent DNA replication |
| GO:0018106 | 4 | 624 | -31.5 | 0.048 | peptidyl-histidine phosphorylation |
| GO:0009252 | 3 | 492 | -25.1 | 0.046 | peptidoglycan biosynthetic process |
| GO:0015893 | 0 | 206 | -12.1 | 0 | drug transport |
| GO:0009292 | 0 | 67 | -3.8 | 0 | genetic transfer |
| GO:0006298 | 0 | 61 | -3.4 | 0 | mismatch repair |

**References:**

1. dos Reis M, Savva R, Wernisch L (2004) Solving the riddle of codon usage preferences: a test for translational selection. Nucleic Acids Res 32: 5036-5044.

2. Sharp PM, Bailes E, Grocock RJ, Peden JF, Sockett RE (2005) Variation in the strength of selected codon usage bias among bacteria. Nucleic Acids Res 33: 1141-1153.

3. Carbone A, Kepes F, Zinovyev A (2005) Codon bias signatures, organization of microorganisms in codon space, and lifestyle. Mol Biol Evol 22: 547-561.
