# Supplementary material for: Translational Selection Is Ubiquitous in Prokaryotes
Source: PLoS Genet. 2010 Jun 24;6(6):e1001004. doi: 10.1371/journal.pgen.1001004 (PMC2891978; doi:10.1371/journal.pgen.1001004)
Supplement: Table S9 — Depletion of OCU genes within the aminoacyl-tRNA synthetases in Bacteria. (0.06 MB DOC) [file pgen.1001004.s015.doc]

**Supporting** **Table S9.** Depletion of OCU genes within the aminoacyl-tRNA synthetases in Bacteria.

| ID | n(OCU) | n(others) | log(pVal) | enrich | description |
| --- | --- | --- | --- | --- | --- |
| GO:0006418 | 261 | 9281 | -205.4 | 0.245 | tRNA aminoacylation for protein translation |
|  |  |  |  |  |  |
| ID | n(OCU) | n(others) | log(pVal) | enrich | description |
| GO:0006423 | 1 | 463 | -21.5 | 0.019 | cysteinyl-tRNA aminoacylation |
| GO:0006428 | 2 | 413 | -17.7 | 0.043 | isoleucyl-tRNA aminoacylation |
| GO:0006425 | 1 | 176 | -7.3 | 0.051 | glutaminyl-tRNA aminoacylation |
| GO:0006427 | 5 | 615 | -24.0 | 0.073 | histidyl-tRNA aminoacylation |
| GO:0006419 | 4 | 448 | -19.1 | 0.073 | alanyl-tRNA aminoacylation |
| GO:0006433 | 3 | 414 | -16.6 | 0.065 | prolyl-tRNA aminoacylation |
| GO:0006431 | 5 | 413 | -14.4 | 0.108 | methionyl-tRNA aminoacylation |
| GO:0006438 | 6 | 416 | -13.7 | 0.128 | valyl-tRNA aminoacylation |
| GO:0006429 | 7 | 413 | -12.8 | 0.150 | leucyl-tRNA aminoacylation |
| GO:0006420 | 15 | 689 | -22.9 | 0.17 | arginyl-tRNA aminoacylation |
| GO:0006432 | 16 | 799 | -22.5 | 0.177 | phenylalanyl-tRNA aminoacylation |
| GO:0006424 | 17 | 845 | -23.7 | 0.178 | glutamyl-tRNA aminoacylation |
| GO:0006437 | 11 | 428 | -10.6 | 0.226 | tyrosyl-tRNA aminoacylation |
| GO:0006436 | 10 | 436 | -11.6 | 0.202 | tryptophanyl-tRNA aminoacylation |
| GO:0006430 | 24 | 506 | -6.9 | 0.408 | lysyl-tRNA aminoacylation |
| GO:0006435 | 21 | 426 | -5.6 | 0.424 | threonyl-tRNA aminoacylation |
| GO:0006434 | 20 | 398 | -5.1 | 0.432 | seryl-tRNA aminoacylation |
| GO:0006422 | 41 | 583 | -5.8 | 0.523 | aspartyl-tRNA aminoacylation |
| GO:0006426 | 46 | 672 | -4.6 | 0.578 | glycyl-tRNA aminoacylation |
| GO:0006421 | 22 | 168 | -0.1 | 1.045 | asparaginyl-tRNA aminoacylation |
