# Supplementary material for: Translational Selection Is Ubiquitous in Prokaryotes
Source: PLoS Genet. 2010 Jun 24;6(6):e1001004. doi: 10.1371/journal.pgen.1001004 (PMC2891978; doi:10.1371/journal.pgen.1001004)
Supplement: Table S10 — Enrichment and depletion of OCU genes within COG groups related to defense from oxidative stress. IDs in the form “COG:xxxx” denote groups from the Clusters of Orthologous Genes database. (0.05 MB DOC) [file pgen.1001004.s016.doc]

**Supporting Table S10.** Enrichment and depletion of OCU genes within COG groups related to defense from oxidative stress. IDs in the form “COG:xxxx” denote groups from the Clusters of Orthologous Genes database.

| COG ID | n(OCU) | n(others) | log(pVal) | enrichment | representative gene in *E. coli* |
| --- | --- | --- | --- | --- | --- |
| *Superoxide dismutase* | |  |  |  |  |
| COG:0605 | 250 | 215 | -111.8 | 4.857 | SodA, SodB (Fe or Mn containing) |
| COG:2032 | 38 | 144 | -5.8 | 1.884 | SodC (Cu/Zn containing) |
| *Catalase* |  |  |  |  |  |
| COG:0753 | 21 | 371 | -4.0 | 0.483 | KatE |
| COG:0376 | 11 | 176 | -1.7 | 0.531 | KatG, KatP |
| *Peroxiredoxins* | |  |  |  |  |
| COG:2077 | 78 | 111 | -25.7 | 3.724 | Tpx |
| COG:0450 | 308 | 223 | -150.2 | 5.241 | AhpC |
| COG:1225 | 137 | 470 | -15.1 | 2.037 | Bcp |
| *Iron-sequestering proteins* | | |  |  |  |
| COG:1528 | 66 | 107 | -19.7 | 3.443 | FtnA, FtnB (ferritin) |
| COG:2193 | 95 | 212 | -20.3 | 2.793 | Bfn (bacterioferritin) |
| COG:0783 | 146 | 199 | -48.8 | 3.820 | Dps (DNA protection during starvation) |
| *Thioredoxin and glutaredoxin* | | |  |  |  |
| COG:0526 | 855 | 2196 | -144.9 | 2.537 | Trx (thioredoxin) |
| COG:0695 | 292 | 246 | -131.9 | 4.904 | GrxA, GrxC (glutaredoxin) |
| COG:0278 | 172 | 64 | -108.9 | 6.581 | YdhD (monothiol glutaredoxin) |

**Reference:**

[1] Tatusov RL, Fedorova ND, Jackson JD, Jacobs AR, Kiryutin B, et al. (2003) The COG database: an updated version includes eukaryotes. BMC Bioinformatics 4: 41.
