# Supplementary material for: Translational Selection Is Ubiquitous in Prokaryotes
Source: PLoS Genet. 2010 Jun 24;6(6):e1001004. doi: 10.1371/journal.pgen.1001004 (PMC2891978; doi:10.1371/journal.pgen.1001004)
Supplement: Text S1 — Supporting methods. (0.15 MB DOC) [file pgen.1001004.s017.doc]

Supporting Information

for manuscript “*Translational selection is ubiquitous in prokaryotes*” by Supek F *et al*.

Text S1: Supporting Methods

[Methods 2](#__RefHeading___Toc254363371)

[Prokaryotic genomes and associated data 2](#__RefHeading___Toc254363372)

[Comparing a classifier to measures of codon usage 3](#__RefHeading___Toc254363373)

[Detecting selection for translational efficiency in genomes 5](#__RefHeading___Toc254363374)

[Assigning optimized codon usage (OCU) labels to individual genes, testing robustness of the assignments 6](#__RefHeading___Toc254363375)

[Tests of enrichment for optimized genes and display of results 7](#__RefHeading___Toc254363376)

[Appendix A. List of mRNA expression datasets. 9](#__RefHeading___Toc254363377)

[Appendix B. List of organisms where translational selection was not detected in previous multi-genome studies. 10](#__RefHeading___Toc254363378)

[References 11](#__RefHeading___Toc254363379)

# Methods

### Prokaryotic genomes and associated data

We have downloaded 621 fully sequenced prokaryotic genomes from the NCBI Entrez Genome FTP site [1] on 21^th^ Jan 2008. Among those, we removed multiple strains of a single species to counter biases toward commonly re-sequenced model organisms or pathogens, such as *Escherichia coli* or *Streptococcus pyogenes*, leaving 461 species. Among the strains, we retained the one best covered by Gene Ontology annotations.

Information about lifestyles of organisms was assembled from two sources: the JCVI Genome Properties database [2], and the NCBI Entrez Microbial Genome Properties [3]. Both databases were curated to remove rare, difficult to interpret, highly redundant or incomplete properties, and to represent all remaining properties of interest as a series of binary (“yes/no” or “low/high”) categorical variables.

We have matched protein coding genes to Gene Ontology (GO) categories [4] of their products using the cross-references provided by the EBI GO Annotation Database [5]; the mapping files used were “gp2protein.geneid” and “gene_association.goa_uniprot”, downloaded on 28^th^ Oct 2008.

The datasets with absolute mRNA abundances in prokaryotes were downloaded from the NCBI Gene Expression Omnibus (GEO), requiring adequate annotation of the experimental platform (recent datasets may still not annotated in such a way to enable automated linking of expression data to standard NCBI gene identifiers. The mRNA abundance data was obtained either using single-channel microarrays (commonly Affymetrix) or two-channel microarrays normalized to genomic DNA. We have manually curated to list to include only datasets where growth conditions can be expected to lead to fast growth of the organism, i.e. the conditions where translational selection would be expected to act – exponential growth phase, rich medium, and no physical, chemical or biological stress for the organism. Depending on the growth conditions, the correlation of gene expression to codon usage might be larger or smaller; this has been shown for yeast [6] and *Escherichia coli* and *Bacillus subtilis* [7]. Finally, we have reduced the list to one dataset per organism, leaving us with 19 datasets with good coverage of various bacteria phyla: Proteobacteria (α, ß, γ and δ), Firmicutes, Actinobacteria and the Thermus/Deinococcus clade. The list of datasets is given as Appendix A in the Supplementary; the set includes four genomes where translational selection was previously regarded as absent (Appendix B in the Supplementary).

Quantitative datasets with protein levels in prokaryotes are generally speaking still scarce and limited to a subset of proteins. However, for *Escherichia coli* in specific, two large-scale quantitative proteomics experiments are available which attempt to measure abundance of a larger number of proteins from the *E. coli* cytoplasm: Ishihama *et al*. [8] provides data on 1103 proteins, and Lu *et al*. [9], 449 proteins. We have filtered the data to retain only the 369 proteins that occur in both studies.

The original data used in all the computations is freely available via the authors’ web site <http://www.adaptome.org/>, as well as from the original sources at NCBI, JCVI and EBI.

### Comparing a classifier to measures of codon usage

We employ a supervised machine learning method – the Random Forest (RF) classifier [10] – to detect signatures of selection acting to optimize translational efficiency of genes. The RF implementation we use is FastRandomForest 0.98 [11] which integrates into the Weka learning environment [12]. RF was our method of choice as it is computationally efficient, robust to noise and handles missing data seamlessly. The 'forest size' parameter ("-I") was set to 1000 trees; other parameters were left at default values.

To construct datasets – one per genome – we declare all genes coding for ribosomal proteins to be the ‘positive class’, assuming ribosomal protein genes to be a class of genes exhibiting strong influences of translational selection on codon usage. All other protein-coding genes are the ‘negative class’.

The RF algorithm produces an ensemble of decision tree classifiers, where each decision tree is constructed by recursively partitioning the data by attribute value tests (forming ‘nodes’) so as to reduce the class entropy in the resulting partitions (‘branches’). In RF, trees are constructed on bootstrap samples of the entire dataset, and choice of attributes at each node is restricted to introduce variability. The final predictions of a RF model are obtained by averaging over individual trees (‘voting’).

Genes shorter than 80 codons, having internal stop codons or having length (in nucleotides) indivisible by three were excluded from computation. A gene is represented by a series of codon frequencies for all degenerate codon families, excluding the stop codons. The frequencies of codons for a single amino acid are adjusted to sum to one, therefore the data does not reflect amino acid frequencies. If an amino acid is absent from a gene product, the amino acid’s codon frequencies are not estimated in any way but are instead represented by a ‘missing value’ symbol in the dataset.

On such datasets, four-fold crossvalidation was run to determine performance of the RF algorithm in discriminating the ribosomal protein genes by their codon frequencies; the area-under-ROC-curve (AUC) score [13] was recorded as implemented in the Weka environment.

The RF classifier was compared to three distance measures for vectors of codon frequencies: (i) “codon bias between gene groups” (CB) is essentially a weighted Manhattan distance employed by Karlin and colleagues [14] for finding ‘predicted highly expressed’ genes in microbial genomes; (ii) the “codon adaptation index” (CAI) is an established surrogate for gene expression under optimal growth conditions of *E. coli* and *S. cerevisiae* [15]; and (iii) “measure independent of length and composition” (MILC) [7] is a corrected χ^2^-type statistic devised to address methodological deficiencies present in other approaches such as CB.

We describe here how the distance measures are computed. Let *G* indicate a gene or a group of genes with codon frequencies *g*(*x*,*y*,*z*) for a codon (*x*,*y*,*z*) normalized such that Σ *g*(*x*,*y*,*z*) = 1, where the sum extends over all codons (*x*,*y*,*z*) translated to amino acid *a*. Let *f*(*x*,*y*,*z*) indicate the codon frequencies of another gene or gene group *F*, again normalized to sum to 1 within each amino acid. Let *p_a_*(*F*) be the amino acid frequencies of the gene/genes in *F*, which sum to 1 over all amino acids.

Distance in the codon frequency space between the genes or gene groups *F* and *G* is estimated by the MILC method as:

$$MILC(F|G)=2\sum_{a} p_{a}(F)\left[\sum_{(x,y,z)=a} f(x,y,z)\cdot\ln\frac{f(x,y,z)}{g(x,y,z)}\right]-\frac{1}{L}\sum_{a} r_{a}+\frac{1}{2}$$

Where *r_a_* is the redundancy class for an amino acid (2 for two-fold degenerate amino acids, 3 for isoleucine, and so on), and *L* is the gene length. The sums should iterate only over the amino acids present in the gene F. Note that the formulae in the original paper [7] contain errors in the equation, where the initial ‘2’ was omitted and the final “1/2” had the wrong sign (- instead of +); for more information, see *Erratum* at <http://www.bioinfo.hr/inca/> .

The ‘codon bias’ measure [14] is computed as:

$$CB(F|G)=\sum_{a} p_{a}(F)\left[\sum_{(x,y,z)=a} \left|f(x,y,z)-g(x,y,z)\right|\right]$$

The codon adaptation index [15] is computed as:

$$CAI(F|G)=\prod_{a} \left[\prod_{(x,y,z)=a} \left(\frac{g(x,y,z)}{g_{\max}((x,y,z)=a)}\right)^{f(x,y,z)}\right]^{p_{a}(F)}$$

where “*g*_max_((*x*,*y*,*z*)=*a*)” denotes the maximum frequency of the codons coding for amino acid *a* in gene group *G*. The ratio *g*/*g*_max_ is called codon ‘relative adaptiveness’ in the original paper of Sharp and Li, and the CAI is a geometric mean of these adaptiveness values of all codons in the gene *F*. In a typical usage scenario of the CAI, the group G would consist of a set of highly expressed genes, sometimes also called a ‘reference set’. In contrast to CB and MILC, CAI decreases with increasing distance.

We have incorporated the three distance measures into a ‘nearest centroid’ type classifier for the Weka environment [12]. In this scheme, a gene is classified as the positive or the negative class depending on the distance to the vector of average codon frequencies within a class (centroid). This procedure is analogous to uses of CB and CAI in the literature. Finally, AUC scores of the RF classifier are compared to AUC scores of nearest centroid classifiers of the distance measures in each of the 461 species.

Note that the distance measures have information about amino acid frequencies at their disposal, while the RF classifier does not. The distance measures have been designed to use the amino acid frequencies as weights on contributions of each codon to the total distance, while RF’s learning process can be thought of as involving implicit estimation of these weights from the correlations of codon frequency data and the class label. The “variable importance” feature of some RF implementations ([16]) might be used to gain insight into these ‘implicit weights’.

As another verification of the utility of the RF classifier in comparison to the distance measures, we compare its output to protein abundance data for *E. coli;* see section “*Prokaryotic genomes and associated data*”. For Figure 1, we have filtered the measurements to retain only the 369 proteins that occur in both studies; we also provide the correlation coefficients (Spearman and Pearson) in Supporting Table S1. For the purposes of this calculation, we set the value of RF's 'forest size' parameter to 5000 trees. Note that there is some discrepancy between the strength of correlation of CAI and CB to expression, as reported by Ishihama *et al*. [8] (Spearman’s ρ=0.5 and 0.53 for CAI and CB, respectively) and as reported here (ρ=0.63 and 0.68 for CAI and CB), which may be due to a use of a different 'reference set' of genes - Ishihama *et al.* [8] do not specify details of how CAI and CB were computed.

### Detecting selection for translational efficiency in genomes

Nucleotide substitution patterns in DNA molecules influence codon frequencies of genes; additionally, the biases in nucleotide substitution patterns may be region, strand, or chromosome specific [17,18]. It is necessary to control for the biases in order to reliably detect influence of translational selection on codon usage.

We encode the information about local nucleotide substitution patterns affecting each gene by computing mononucleotide and dinucleotide frequencies in the non-coding regions of DNA neighboring the translated part of the gene. Genes for functional RNA molecules such as tRNA and rRNA are also treated as coding and thus do not contribute toward mono- and di-nucleotide frequencies of intergenic DNA. The size of the neighborhood window was set to either 5, 10 or 20 kilobases upstream from the gene’s start codon, and 5, 10 or 20 kilobases downstream from the stop codon. The window size of 10 kb upstream + 10 kb downstream guarantees that in 99% of the genomes (457 out of 461), 99% of the genes have at least 142 non-coding nucleotides available for estimation of mono- and di-nucleotide frequencies (Supporting Table S2). We have re-run the analysis described below with the three values of the ‘window size’ parameter and combined the results into a consensus set at a later stage (see below).

To detect if selection for translational efficiency acts on a genome, we employ the following procedure (described graphically in Fig. 1). The RF classifier is first trained to distinguish ribosomal protein genes (‘positive class’) based solely on the mono- and di-nucleotide frequencies of genes’ neighboring non-coding DNA within a given window size. Fifty runs of four-fold crossvalidation are used to estimate the accuracy of the classifier using the area-under-ROC-curve (AUC) [13] score, and the AUC for each of the 50 runs of crossvalidation is recorded.

The procedure is then repeated for a second time, however now the codon frequencies are also included in the dataset for the RF classifier training, in addition to description of the intergenic regions. The AUC scores are again recorded for each of the 50 runs of crossvalidation. To determine if selection for translational efficiency acts on the genome, the sign test [19] is used to compare 50 AUC scores obtained without codon frequencies to 50 AUC scores obtained with codon frequencies, for each genome (Fig. 1). If the AUC score exhibits a consistent increase over 50 runs of crossvalidation, the introduction of codon frequencies improves the ability to discriminate ribosomal protein genes, providing evidence that translational selection acts on this specific genome. A summary of results for window size 10k is presented in Supporting Table S3. The weakest result is observed in the bacterium *Saccharophagus degradans 2-40*, and is the only genome where the change in AUC scores is not statistically significant at *p*<10^-3^. The majority of examined prokaryotes (457 of 461) have the sign test *p*<10^-9^. The results are qualitatively equivalent with window sizes 5k and 20k (not shown), with 460 and 459 (of 461) genomes, respectively, having sign test *p<*10^-9^. Note that with window size of 5k, the *Saccharophagus degradans 2-40* genome does exhibit a statistically significant increase in AUC with introduction of codon frequencies to the classifiers (*p*=1.19·10^-5^), although this still is the least significant *p*-value among the 461 genomes.

### Assigning optimized codon usage (OCU) labels to individual genes, testing robustness of the assignments

To declare a gene to be under influence of translational selection, during the procedure described above which involves two rounds of RF classifier training – without and with codon frequencies – the per-gene probabilities of belonging to positive class are recorded for each of the 50 runs of crossvalidation.

The per-gene probabilities are then compared between the two rounds of crossvalidation. A sign test [19] is used to determine if an increase in probability occurs more frequently than expected by chance; if it does, the gene is labeled as having optimized codon usage (OCU). At this point, we combine the OCU assignments obtained with the three values of the window size parameter (5, 10 and 20 kilobases) into a consensus set, by determining the median *p*-value of sign tests of the three window sizes for each gene. The consensus data is used in all further computations, with exception of the non-coding DNA survey in Supplementary Table 1 and the RF robustness test in Supplementary Table 4 which were performed using a window size of 10 kilobases.

We have set the sign test threshold *p*-value to 10^-15^; this *p*-value corresponds to exactly 50 out of 50 wins for the dataset with codon frequencies, and consequently zero wins for the dataset without codon frequencies. This sign test *p*-value should be regarded as somewhat optimistic because the repeated runs of crossvalidation are not independent, being based on repeated sampling from the same set of genes. To obtain a conservative estimate, we employed a corrected paired *t*-test [72] intended for comparison of classification algorithms using repeated runs of crossvalidation. Note that we actually compare RF models derived from specific dataset, and not the different variants of the underlying RF algorithm itself, and therefore this *p*-value would be pessimistic for our experimental setup [72]. After this corrected *t*-test, the median *p*-value for OCU genes in 10 representative genomes (Text S2) was 6·10^-6^, while for 95% of the OCU genes, *p* < 2·10^-3^*.* For the difference of with/without codons AUC score, the median *p*-value for all 461 genomes obtained by the corrected paired *t*-test was *p*=10^-13^; for 95% of the genomes, *p* < 4·10^-5^ (compare to median *p*=10^-15^ by sign test).

We further test the robustness of the genes’ OCU assignments by performing several computational experiments; all results are supplied in tables within Text S2, while a description of the test procedures and a summary of the findings is given here. First experiment is a simulation to see if a methodological bias exists where changes in the positive-to-negative class size ratio would affect frequency of OCU labels (Text S2). We demonstrate the extent of such changes to be relatively minor, and insufficient to explain the correlation between genome size and % OCU described in Results (Figure 3).

The second experiment verifies if an outlier in the positive class – a RP gene with atypical codon usage – would affect OCU assignments. We found that our RF-classifier based methodology for OCU detection is remarkably robust (Text S2) to such errors in annotation that could stem, for instance, from pseudogenes or from the uncommon events of RP gene horizontal transfer [20]. This result is in accord with previous work [10,21] that established that RF and the ‘bagging’ technique it is based on are not overly sensitive to ‘output noise’ by using simulations where class labels (here: RP/non-RP) are altered, basically creating outliers.

We also examine to what degree the choice of the window size parameter (5, 10 or 20 kilobases) influences the decisions about genes’ OCU/non-OCU label. The results generally agree well between window sizes (Text S2), with on average 90.7% of the genes retaining their OCU/non-OCU label when the window size changes from 10 kb to 5 or 20 kb (assuming independence of labels, 75.9% agreement would be expected). Moreover, the consensus set further improves on this, offering on average 95.2% agreement in genes’ OCU/non-OCU labels with the individual 5, 10 or 20 kb label sets (here, 76.5% agreement would be expected assuming independence of labels).

The datasets derived from the 461 genomes exhibit highly imbalanced class sizes; the positive class (ribosomal protein genes) contains on average 54.4 genes, while the average size of the negative class is much larger, at 3025.7 genes. Moreover, the positive-to-negative class ratio is quite variable among genomes as the number of ribosomal protein genes is practically constant, and genomes range in size from 461 to 9178 genes (genes ≥80 codons). To test the behavior of the RF classifier-based methodology for detection of OCU in this difficult situation of highly imbalanced and highly variable class proportions, we conduct a computational simulation. For ten representative genomes, we remove half of the negative class instances at random to induce a 2-fold increase in the positive-to-negative class ratio, and observe the effect on % OCU genes (Text S2). The 2x changes in the class ratios have induced much smaller changes in the % OCU within the negative class, on average 1.16x (range: 1.03x - 1.35x), indicating the RF-based OCU detection methodology is robust to changes in class proportions, even with originally highly imbalanced classes.

### Tests of enrichment for optimized genes and display of results

After obtaining lists of genes estimated to be optimized for translational selection, we use a series of Fisher’s exact tests for association of two categorical variables [22] to describe distribution of OCU genes along Gene Ontology [4] functional categories or COG orthologous groups [23] in all analyzed genomes, and in genomes grouped by environmental and phenotypic contexts (lifestyles) as defined in the JCVI Genome Properties [2], and the NCBI Entrez Microbial Genome Properties ([24]) databases.

To this end, we perform two kinds of tests.

‘Test A’ operates across all genes in all genomes, and compares distribution of optimized genes within a GO category to the distribution of optimized genes outside the GO category, and is iterated over all GO categories. ‘Test B’ operates within a single GO category / COG group, and compares organisms with a specific lifestyle to all organisms which are known not to possess the lifestyle. Test B is iterated over all possible combinations of GO/COG and lifestyle.

We exclude from testing GO categories and COG groups with less than 15 genes in total (test A), or GO categories and COG groups with less than 15 genes within an organism lifestyle group (test B).

In Archaea, 25022 tests remain after the filtering (3335 tests A and 21687 tests B). We set the threshold *p*-value to 10^-3^, yielding an estimated 25.0 false positives out of the 1597 total positives (1.6% false discovery rate).

In Bacteria, 141428 tests remain after filtering small categories (7626 tests A and 133802 tests B). Here, we also set the threshold *p*-value to 10^-3^, yielding 141.4 false positives out of the 8434 total positives (1.7% false discovery rate).

All tests passing the criteria for statistical significance were additionally screened to retain only tests with a sufficient magnitude of enrichment or depletion of optimized genes (>1.50x or <0.67x) between GO categories / COGs (test A) or organism groups (test B). All test results passing the thresholds for gene group size, for statistical significance and for magnitude of enrichment/depletion are available in Supplementary Dataset 2, or from the authors’ Web site at <http://www.adaptome.org/>.

Figure 6 in the manuscript was generated in the following manner: threshold *p*-value was set to a more strict value of p=10^-15^. For the OCU-enriched (green) GO categories, at least 2.0x enrichment was required, leaving 241 GO categories. As we were less interested in the OCU-depleted (red) GO categories, half of that number (120) most strongly depleted GO categories were considered for visualization, corresponding to a depletion of 0.051x or stronger. To summarize the results, the 335 GO categories were then subjected to an semi-automated redundancy elimination procedure where semantic similarity of all possible pairs of categories was quantified per SimRel method [25], and then one member of the currently most similar pair was recursively deleted from the set, while in most cases keeping the category with the strongest *p*-value. This specific approach to reducing redundancy in lists of GO categories was implemented in the REViGO web server at <http://revigo.irb.hr/>, as well as several different approaches to visualizing such lists, such as the graph visualization described below.

Results of the elimination procedure were curated manually to achieve a more informative summary; they are available in full as Supporting Dataset S2, or from the authors’ Web site at <http://www.adaptome.org/>. We visualized the data as a graph in Cytoscape [26] where initial placement of the discs representing GO categories was optimized by the “Edge-Weighted Spring Embedded” layout algorithm, with edge weights in the graph representing semantic similarities between the GO categories. We took the same approach to summarization of results for the ‘molecular function’ ontology in Supporting Table S7, again starting from the same set of 230 most strongly OCU-enriched, and 115 most strongly OCU-depleted GO categories.

Appendix A. List of mRNA expression datasets.

**Appendix A.** List of mRNA expression datasets; the NCBI GEO Series ID is given (“GSExxxx”), together with the GEO Samples (“GSMxxxx”) from the series that were averaged to obtain final expression level.

**(A) absolute mRNA signal intensities, from single-channel microarrays (commonly Affymetrix)**

*Pseudomonas aeruginosa*: GSE4026 (GSM92178, GSM92179, GSM92182, GSM92183, GSM92186, GSM92187)

*Staphylococcus aureus* Mu50: GSE2728 (GSM52706, GSM52707, GSM52755, GSM52724, GSM52725, GSM52727, GSM52744, GSM52745, GSM52746)

*Bifidobacterium longum*: GSE5865 (GSM136748, GSM136749, GSM136750, GSM136751, GSM136752)

*Bradyrhizobium japonicum*: GSE12491 (GSM210242, GSM210243, GSM210244, GSM210245)

*Haemophilus influenzae*: GSE5061 (GSM114031, GSM114032, GSM114033)

*Lactobacillus plantarum*: GSE11383 (GSM287549, GSM287553, GSM287554, GSM287555, GSM287556, GSM287559)

*Listeria monocytogenes*: GSE3247 (GSM73161, GSM73162, GSM73164, GSM73165, GSM73166, GSM73167, GSM73168, GSM73169, GSM73170, GSM73171, GSM73172, GSM73173, GSM73174, GSM73175, GSM73176, GSM73177, GSM73178, GSM73179, GSM73180, GSM73181, GSM73182, GSM73183, GSM73184, GSM73185)

*Nitrosomonas europaea*: GSE10507 (GSM265514, GSM265516, GSM265518, GSM265521, GSM265523, GSM265524)

*Pseudomonas syringae tomato* DC3000: GSE4848 (GSM109003, GSM109007, GSM109009, GSM109011, GSM109012, GSM109014)

*Rhodobacter sphaeroides 2.4.1*: GSE12269 (GSM308084, GSM308085, GSM308086, GSM308087, GSM308088, GSM308089, GSM308090, GSM308091, GSM308092, GSM308093, GSM308094, GSM308095, GSM308096, GSM308097, GSM308098, GSM308099, GSM308100, GSM308101, GSM308102, GSM308103, GSM308104, GSM308105, GSM308106, GSM308107)

*Rhodopseudomonas palustris* CGA009: GSE6221 (GSM143539, GSM143540, GSM143541, GSM143542, GSM143543, GSM143544)

*Streptococcus mutans*: GSE6973 (GSM160647, GSM160648, GSM160649, GSM160650, GSM160651, GSM160652, GSM160653, GSM160654, GSM160655, GSM160656, GSM160657, GSM160658, GSM160659, GSM160660, GSM160661, GSM160662, GSM160663, GSM160664, GSM160665, GSM160666, GSM160667, GSM160668, GSM160669, GSM160670, GSM160671, GSM160672, GSM160673, GSM160674, GSM160675, GSM160676, GSM160677, GSM160678, GSM160679, GSM160680, GSM160681, GSM160682, GSM160683, GSM160684, GSM160685, GSM160686, GSM160687, GSM160688, GSM160689, GSM160690, GSM160691, GSM160692, GSM160693, GSM160694)

*Thermus thermophilus HB8*: GSE10368 (GSM261559, GSM261569, GSM261594, GSM261560, GSM261570, GSM261595)

*Bacillus subtilis*: GSE11937 (GSM299792, GSM299793, GSM299794, GSM299795)

*Escherichia coli K12*: GSE13982 (GSM351280,GSM351282,GSM351295,GSM351297)

**(B) absolute mRNA signal intensities, from dual-channel microarrays normalized to genomic DNA**

*Streptomyces coelicolor*: GSE7172 (GSM172822, GSM172823, GSM172826, GSM172830)

*Desulfovibrio vulgaris Hildenborough*: GSE4447 (GSM101160, GSM101161, GSM101162, GSM101166, GSM101167, GSM101168, GSM101325, GSM101326, GSM101330, GSM101331, GSM101332, GSM101333)

*Salmonella typhimurium LT2*: GSE4631 (GSM103442, GSM103443, GSM103444, GSM103445, GSM103450, GSM103757, GSM103758, GSM103759)

Appendix B. List of organisms where translational selection was not detected in previous multi-genome studies.

**Appendix B.** List of organisms where translational selection was not detected in at least 2 out of 3 previous multi-genome studies [31,32,33]. Eleven genomes marked with an asterisk were claimed to lack translational selection in all three studies.

*Aeropyrum pernix*

*Borrelia burgdorferi* *

*Buchnera aphidicola Sg* *

*Campylobacter jejuni* RM1221

*Caulobacter crescentus*

*Chlamydia muridarum*

*Chlamydia trachomatis* *

*Chlamydophila caviae*

*Chlamydophila pneumoniae* AR39

*Chlorobium tepidum* TLS *

*Fusobacterium nucleatum*

*Halobacterium sp*

*Helicobacter pylori* 26695 *

*Methanopyrus kandleri*

*Mycobacterium tuberculosis* CDC1551

*Mycoplasma genitalium*

*Mycoplasma pneumoniae*

*Mycoplasma pulmonis*

*Neisseria meningitidis* MC58

*Nitrosomonas europaea*

*Nostoc sp.*

*Pseudomonas aeruginosa*

*Pyrobaculum aerophilum*

*Pyrococcus horikoshii*

*Ralstonia solanacearum*

*Rickettsia conorii* *

*Rickettsia prowazekii*

*Sinorhizobium meliloti*

*Streptomyces avermitilis*

*Streptomyces coelicolor* *

*Thermoplasma acidophilum*

*Thermosynechococcus elongatus* *

*Treponema pallidum* *

*Tropheryma whipplei* Twist

*Wigglesworthia brevipalpis* *

*Xanthomonas campestris*

*Xanthomonas citri*

*Xylella fastidiosa* Temecula1 *

# References

1. ftp://ftp.ncbi.nih.gov/genomes/Bacteria/

2. Selengut JD, Haft DH, Davidsen T, Ganapathy A, Gwinn-Giglio M, et al. (2007) TIGRFAMs and Genome Properties: tools for the assignment of molecular function and biological process in prokaryotic genomes. Nucleic Acids Res 35: D260-264.

3. http://www.ncbi.nlm.nih.gov/genomes/lproks.cgi

4. Ashburner M, Ball CA, Blake JA, Botstein D, Butler H, et al. (2000) Gene ontology: tool for the unification of biology. The Gene Ontology Consortium. Nat Genet 25: 25-29.

5. http://www.ebi.ac.uk/GOA/

6. Wagner A (2000) Inferring lifestyle from gene expression patterns. Mol Biol Evol 17: 1985-1987.

7. Supek F, Vlahovicek K (2005) Comparison of codon usage measures and their applicability in prediction of microbial gene expressivity. BMC Bioinformatics 6: 182.

8. Ishihama Y, Schmidt T, Rappsilber J, Mann M, Hartl FU, et al. (2008) Protein abundance profiling of the Escherichia coli cytosol. BMC Genomics 9: 102.

9. Lu P, Vogel C, Wang R, Yao X, Marcotte EM (2007) Absolute protein expression profiling estimates the relative contributions of transcriptional and translational regulation. Nat Biotechnol 25: 117-124.

10. Breiman L (2001) Random forests. Machine Learning 45: 5-32.

11. http://fast-random-forest.googlecode.com/

12. Witten IH, Frank E (2005) Data Mining: Practical machine learning tools and techniques. San Francisco: Morgan Kaufmann.

13. Fawcett T (2006) An introduction to ROC analysis. Pattern Recognition Letters 27: 861-874.

14. Karlin S, Mrazek J (2000) Predicted highly expressed genes of diverse prokaryotic genomes. J Bacteriol 182: 5238-5250.

15. Sharp PM, Li WH (1987) The codon Adaptation Index--a measure of directional synonymous codon usage bias, and its potential applications. Nucleic Acids Res 15: 1281-1295.

16. http://www.stat.berkeley.edu/~breiman/RandomForests/cc_home.htm

17. Daubin V, Perriere G (2003) G+C3 structuring along the genome: a common feature in prokaryotes. Mol Biol Evol 20: 471-483.

18. Rocha EP (2004) The replication-related organization of bacterial genomes. Microbiology 150: 1609-1627.

19. McDonald JH (2008) Sign test. Handbook of Biological Statistics. Baltimore: Sparky House Publishing. pp. 185-189.

20. Chen K, Roberts E, Luthey-Schulten Z (2009) Horizontal gene transfer of zinc and non-zinc forms of bacterial ribosomal protein S4. BMC Evol Biol 9: 179.

21. Dietterich TG (2000) An experimental comparison of three methods for constructing ensembles of decision trees: Bagging, boosting, and randomization. Machine Learning 40: 139-157.

22. McDonald JH (2008) Fisher's exact test of independence. Handbook of Biological Statistics. Baltimore: Sparky House Publishing. pp. 64-68.

23. Tatusov RL, Fedorova ND, Jackson JD, Jacobs AR, Kiryutin B, et al. (2003) The COG database: an updated version includes eukaryotes. BMC Bioinformatics 4: 41.

24. http://www.ncbi.nlm.nih.gov/genomes/lproks.cgi

25. Schlicker A, Domingues FS, Rahnenfuhrer J, Lengauer T (2006) A new measure for functional similarity of gene products based on Gene Ontology. BMC Bioinformatics 7: 302.

26. Shannon P, Markiel A, Ozier O, Baliga NS, Wang JT, et al. (2003) Cytoscape: a software environment for integrated models of biomolecular interaction networks. Genome Res 13: 2498-2504.

27. Breiman L, Cutler A (2001) http://www.stat.berkeley.edu/~breiman/RandomForests/cc_home.htm#outliers.

28. Molina N, van Nimwegen E (2008) Universal patterns of purifying selection at noncoding positions in bacteria. Genome Res 18: 148-160.

29. Neuhauser M, Senske R (2004) The Baumgartner-Weiss-Schindler test for the detection of differentially expressed genes in replicated microarray experiments. Bioinformatics 20: 3553-3564.

30. Chan PP, Lowe TM (2009) GtRNAdb: a database of transfer RNA genes detected in genomic sequence. Nucleic Acids Res 37: D93-97.

31. dos Reis M, Savva R, Wernisch L (2004) Solving the riddle of codon usage preferences: a test for translational selection. Nucleic Acids Res 32: 5036-5044.

32. Sharp PM, Bailes E, Grocock RJ, Peden JF, Sockett RE (2005) Variation in the strength of selected codon usage bias among bacteria. Nucleic Acids Res 33: 1141-1153.

33. Carbone A, Kepes F, Zinovyev A (2005) Codon bias signatures, organization of microorganisms in codon space, and lifestyle. Mol Biol Evol 22: 547-561.
