# Supplementary material for: Translational Selection Is Ubiquitous in Prokaryotes
Source: PLoS Genet. 2010 Jun 24;6(6):e1001004. doi: 10.1371/journal.pgen.1001004 (PMC2891978; doi:10.1371/journal.pgen.1001004)
Supplement: Text S2 — Tests of robustness of the RF classifier-based methodology for assigning OCU labels to genes. (0.17 MB DOC) [file pgen.1001004.s018.doc]

Supporting Information

for manuscript “*Translational selection is ubiquitous in prokaryotes*” by Supek F *et al*.

**Text S2: Tests of robustness of the RF classifier-based methodology for assigning OCU labels to genes.**

[Text S2, Table 1. Robustness to presence of horizontally transferred (HT) segments in DNA. 2](#__RefHeading___Toc254277158)

[Text S2, Table 2. *p*-values (by corrected t-test) for OCU assignments. 3](#__RefHeading___Toc254277159)

[Text S2, Table 3. Robustness to changing size of window for collecting non-coding DNA. 4](#__RefHeading___Toc254277160)

[Text S2, Table 4. Robustness to changing proportions of the positive and negative class. 5](#__RefHeading___Toc254277161)

[Text S2, Table 5. Robustness to outliers with atypical codon usage in the ribosomal protein gene set. 6](#__RefHeading___Toc254277162)

[Text S2, Table 6. Robustness to selection acting on a non-coding DNA. 7](#__RefHeading___Toc254277163)

Text S2, Table 1. Robustness to presence of horizontally transferred (HT) segments in DNA.

**Text S2, Table 1.** Robustness of the RF classifier-based methodology to presence of horizontally transferred (HT) segments in DNA. We used the IslandViewer database [1] that detects HT segments using three different algorithms, combined predictions of all three algorithms by a logical 'OR' and masked all intergenic DNA within these segments and excluded all genes overlapping the HT segments, thus obtaining the “Filtered data”. Re-running the complete computational workflow yielded the “%OCU-Filtered data” column. “Agreement” is defined as the percentage of genes that do not change their OCU/non-OCU status between the “Original” and the “Filtered” data, or between subsequent runs on the “Original” data (quantifying variation introduced by our method).

|  | # of genes (>80 codons) | | | % OCU | | agreement with original | |
| --- | --- | --- | --- | --- | --- | --- | --- |
| organism | Original data | In HT segments | Filtered data | Original data * | Filtered data | Between runs | Filtered data |
| Bacillus subtilis | 3880 | 342 | 3538 | 7.72% | 9.24% | 98.24% | 96.95% |
| Borrelia burgdorferi | 1385 | 22 | 1363 | 22.30% | 22.16% | 93.71% | 92.81% |
| Deinococcus radiodurans | 3099 | 84 | 3015 | 10.65% | 10.75% | 97.64% | 97.45% |
| Escherichia coli K12 | 3959 | 389 | 3570 | 7.11% | 7.39% | 98.49% | 98.26% |
| Halobacterium sp. | 2408 | 141 | 2267 | 18.75% | 20.34% | 94.35% | 93.30% |
| Helicobacter pylori 26695 | 1478 | 60 | 1418 | 18.69% | 18.76% | 95.71% | 95.98% |
| Mycoplasma genitalium | 465 | 0 ** | 465 | 16.34% | 16.34% | 94.84% | 100.00% |
| Pelagibacter ubique HTCC1062 | 1293 | 16 | 1277 | 19.19% | 20.28% | 95.36% | 94.05% |
| Pseudomonas aeruginosa | 5429 | 121 | 5308 | 5.97% | 6.25% | 98.64% | 98.55% |
| Streptomyces coelicolor | 7801 | 392 | 7409 | 6.59% | 6.94% | 98.46% | 97.98% |

* The "%OCU - Original data" column has slightly different values than the "% OCU - Consensus" column in Text S2, Table 3 as % OCU is here measured only on the genes present in the "Filtered" dataset, enabling a more exact comparison.

** The *Mycoplasma genitalium* genome has no predicted HT segments in the IslandViewer database.

**Reference:**

[1] Langille MGI, Brinkman FSL (2009) IslandViewer: an integrated interface for computational identification and visualization of genomic islands. Bioinformatics 25(5):664-665.

Text S2, Table 2. p-values (by corrected t-test) for OCU assignments.

**Text S2, Table 2.** *p*-values (by corrected paired *t*-test) for OCU assignments. The OCU labels were originally obtained by the sign test at *p*=10^-15^. The corrected paired *t*-test [1] was originally designed for comparison of classification algorithms using repeated runs of crossvalidation.

| Genome | # genes | % OCU | median *p*-value of OCU genes by corrected *t*-test [1] | 95th percentile of *p*-values of OCU genes by corrected *t*-test [1] |
| --- | --- | --- | --- | --- |
| *Bacillus subtilis* | 3880 | 7.72% | 3.0E-06 | 2.1E-03 |
| *Borrelia burgdorferi* | 1385 | 22.30% | 4.2E-05 | 7.8E-03 |
| *Deinococcus radiodurans* | 3099 | 10.65% | 1.3E-06 | 1.5E-03 |
| *Escherichia coli K12* | 3959 | 7.11% | 3.0E-07 | 2.4E-03 |
| *Halobacterium sp.* | 2408 | 18.75% | 8.5E-05 | 8.5E-03 |
| *Helicobacter pylori 26695* | 1478 | 18.69% | 5.3E-06 | 1.2E-03 |
| *Mycoplasma genitalium* | 465 | 16.34% | 1.5E-07 | 3.4E-04 |
| *Pelagibacter ubique HTCC1062* | 1293 | 19.19% | 6.3E-06 | 2.2E-03 |
| *Pseudomonas aeruginosa* | 5429 | 5.97% | 2.5E-05 | 5.6E-03 |
| *Streptomyces coelicolor* | 7801 | 6.59% | 3.6E-05 | 3.3E-03 |
| *Median of 10 shown genomes* | *2754* | *13.50%* | *5.8E-06* | *2.3E-03* |

**Reference:**

[1] Nadeau C, Bengio Y (2003) Inference for the generalization error. Machine Learning 52: 239-281.

Text S2, Table 3. Robustness to changing size of window for collecting non-coding DNA.

**Text S2, Table 3.** A test for robustness of the RF classifier-based methodology with regard to changing window size for determining mono- and di-nucleotide frequencies in non-coding DNA. “Win05k” implies that the mono- and di-nucleotide frequencies were computed from the non-coding DNA at most 5 kilobases upstream from a gene’s start codon, and at most 5 kilobases downstream from the gene’s stop codon (total span: 10 kilobases + length of gene); an analogous definition holds true for “win10k” and “win20k”. “Agreement” is defined as the percentage of genes that do not change their OCU/non-OCU status as window size is changed. “Consensus” uses the median sign test *p*-values (see Methods or SI Text) of the three window sizes to determine OCU/non-OCU status of a gene.

|  |  | % OCU genes in genome | | | | agreement with win10k | | agreement with consensus | | |
| --- | --- | --- | --- | --- | --- | --- | --- | --- | --- | --- |
|  | gene count * | win05k | win10k | win20k | **consensus** | win05k | win20k | win05k | win10k | win20k |
| *Bacillus subtilis* | 3814 | 7.94% | 9.12% | 10.44% | **8.44%** | 95.4% | 94.8% | 97.7% | 97.6% | 97.2% |
| *Borrelia burgdorferi* | 1384 | 22.62% | 21.46% | 26.16% | **21.97%** | 84.8% | 84.6% | 92.1% | 92.7% | 91.9% |
| *Deinococcus radiodurans* | 3096 | 10.50% | 11.50% | 12.98% | **10.85%** | 94.4% | 92.7% | 97.1% | 97.3% | 95.4% |
| *Escherichia coli K12* | 3914 | 7.89% | 7.97% | 8.25% | **7.49%** | 96.0% | 95.6% | 97.9% | 98.1% | 97.5% |
| *Halobacterium sp.* | 2391 | 18.11% | 19.87% | 23.59% | **19.24%** | 86.4% | 85.9% | 91.8% | 94.7% | 91.2% |
| *Helicobacter pylori 26695* | 1470 | 17.07% | 20.34% | 24.15% | **19.18%** | 87.1% | 86.4% | 92.9% | 94.2% | 92.2% |
| *Mycoplasma genitalium* | 465 | 18.28% | 17.42% | 21.94% | **16.34%** | 88.4% | 83.0% | 95.1% | 93.3% | 89.7% |
| *Pelagibacter ubique HTCC1062* | 1292 | 20.20% | 19.89% | 21.90% | **19.12%** | 86.8% | 86.2% | 92.7% | 94.1% | 92.1% |
| *Pseudomonas aeruginosa* | 5426 | 6.27% | 6.25% | 6.78% | **6.19%** | 96.7% | 96.4% | 98.3% | 98.4% | 97.9% |
| *Streptomyces coelicolor* | 7772 | 6.86% | 7.18% | 7.48% | **6.87%** | 96.5% | 95.9% | 98.2% | 98.3% | 97.5% |

* Genes shorter than 80 codons are excluded from all computations, and therefore also from this count.

Text S2, Table 4. Robustness to changing proportions of the positive and negative class.

**Text S2, Table 4.** A test for robustness of the RF classifier-based methodology with regard to changing proportions of the positive (ribosomal protein genes) and negative (other protein genes) class. The simulated genomes have 50% of the negative class instances removed at random, corresponding to a two-fold increase in the positive vs. negative class ratio. The size of the positive class is kept constant in the simulation.

|  | actual genome | | | simulated genomes (*n* = 5) with 50% of negative class removed | | | | | |
| --- | --- | --- | --- | --- | --- | --- | --- | --- | --- |
| Genome | gene count * | class ratio (pos:neg) | % OCU** | gene count * | class ratio (pos:neg) | %OCU ** | | | %OCU fold change |
|  |  |  |  |  |  | avg | min | max |  |
| *Bacillus subtilis* | 3814 | 1 : 83.8 | 8.65 % | 1929 | 1 : 41.9 | 10.08% | 9.77% | 10.24% | 1.17 x |
| *Borrelia burgdorferi* | 1384 | 1 : 29.1 | 21.67 % | 715 | 1 : 14.5 | 25.08% | 24.66% | 25.56% | 1.16 x |
| *Deinococcus radiodurans* | 3096 | 1 : 67.8 | 10.98 % | 1570 | 1 : 33.9 | 12.65% | 11.93% | 12.98% | 1.15 x |
| *Escherichia coli K12* | 3914 | 1 : 88.0 | 7.57 % | 1979 | 1 : 44.0 | 8.26% | 7.75% | 8.63% | 1.09 x |
| *Halobacterium sp.* | 2391 | 1 : 51.0 | 19.70 % | 1218 | 1 : 25.5 | 26.14% | 25.60% | 27.39% | 1.33 x |
| *Helicobacter pylori 26695* | 1470 | 1 : 34.9 | 20.01 % | 755 | 1 : 17.4 | 21.04% | 19.75% | 22.41% | 1.05 x |
| *Mycoplasma genitalium* | 465 | 1 : 9.8 | 16.82 % | 254 | 1 : 4.9 | 17.25% | 14.69% | 18.96% | 1.03 x |
| *Pelagibacter ubique HTCC1062* | 1292 | 1 : 27.7 | 19.57 % | 668 | 1 : 13.8 | 20.39% | 19.58% | 21.35% | 1.04 x |
| *Pseudomonas aeruginosa* | 5426 | 1 : 122.3 | 5.82 % | 2735 | 1 : 61.2 | 7.87% | 7.58% | 7.99% | 1.35 x |
| *Streptomyces coelicolor* | 7772 | 1 : 168.0 | 6.90 % | 3909 | 1 : 84.0 | 8.66% | 8.15% | 9.01% | 1.25 x |

* Genes shorter than 80 codons are excluded from all computations, and therefore also from this count.

** Here, % OCU is measured among the non-ribosomal protein genes only (i.e. negative class). OCU status of genes was determined using a single window size for the mono- and di-nucleotide frequencies, 10 kilobases upstream from the start codon of a gene, and 10 kilobases downstream from the stop codon, see Methods, and Supplementary Table 3.

Text S2, Table 5. Robustness to outliers with atypical codon usage in the ribosomal protein gene set.

**Text S2, Table 5.**  A test for robustness of the Random Forest (RF) classifier‐based methodology with regard to outliers (genes with atypical codon usage) in the ribosomal protein gene set. The „original run“ represents the data used throught the paper. The „repeated run“ means the RF-based computational procedure for detecting OCU genes was run again on these 12 genomes with no changes in the input parameters except the 'seed' for initializing the random number generator; this was done to quantify variation introduced by our methology for OCU detection. The „strongest outlier removed“ are the datasets with one ribosomal protein (RP) gene removed – the one that deviated most from the codon usage of RP genes, as quanified by the 'outlier score' of RF classifier [27]. Median 'outlier score' among all 461 genomes is 9.82, range: 2.73 to 42.02.

|  | % OCU in genome | | | *p*-value for difference in AUC scores (sign test) | agreement with original run | | |
| --- | --- | --- | --- | --- | --- | --- | --- |
| organism, outlier gene information | original  run | repeated  run | strongest outlier  removed |  | repeated run | strongest outlier removed | difference |
| *Acinetobacter baumannii* ATCC 17978  outlier GeneID 4918765, score 39.0  COG0254, “50s ribosomal protein l31 type b” | 8.35% | 8.23% | 7.92% | 8.88·10^-16^ | 98.40% | 98.10% | -0.31% |
| *Bacillus cereus* ATCC 10987  outlier GeneID 2751057, score 39.3  no COG, “ribosomal protein l5 domain protein” | 7.63% | 7.49% | 7.63% | 4.53·10^-14^ | 98.44% | 98.38% | -0.06% |
| *Brucella ovis*  outlier GeneID 5202819, score 40.8  COG4233, “ribosomal protein s7” | 11.33% | 11.67% | 11.44% | 8.88·10^-16^ | 97.11% | 96.66% | -0.45% |
| *Lactobacillus casei* ATCC 334  outlier GeneID 4419984, score 40.2  COG1358, “ribosomal protein hs6-type (s12/l30/l7a)” | 10.57% | 10.57% | 9.66% | 8.88·10^-16^ | 97.93% | 97.66% | -0.28% |
| *Lactobacillus reuteri* F275  outlier GeneID 5188707, score 39.4  COG1358, “ribosomal protein l7ae/l30e/s12e/gadd45” | 10.55% | 10.27% | 10.04% | 8.88·10^-16^ | 98.27% | 97.94% | -0.33% |
| *Polaromonas* JS666  outlier GeneID 4012400, score 39.5  no COG, “60s ribosomal protein l19” | 13.43% | 13.13% | 13.41% | 8.88·10^-16^ | 97.31% | 97.47% | 0.17% |
| *Propionibacterium acnes* KPA171202  outlier GeneID 2932330, score 39.0  COG0199, “30s ribosomal protein s14” | 14.46% | 14.78% | 14.01% | 8.88·10^-16^ | 98.05% | 97.82% | -0.23% |
| *Saccharopolyspora erythraea* NRRL 2338  outlier GeneID 4940614, score 40.8  COG0199, “30s ribosomal protein s14” | 10.08% | 10.24% | 10.03% | 8.88·10^-16^ | 97.23% | 97.14% | -0.09% |
| *Salinispora tropica* CNB-440  outlier GeneID 5060855, score 40.3  COG0522, “30s ribosomal protein s4” | 12.57% | 12.25% | 12.32% | 8.88·10^-16^ | 97.09% | 96.57% | -0.52% |
| *Shewanella sediminis* HAW-EB3  outlier GeneID 5613367, score 39.3  no COG, “s23 ribosomal” | 8.60% | 8.60% | 8.37% | 8.88·10^-16^ | 98.40% | 98.35% | -0.05% |
| *Staphylococcus aureus* COL  outlier GeneID 3238344, score 42.0  COG1358, “30s ribosomal protein l7 ae” | 11.02% | 10.81% | 10.72% | 8.88·10^-16^ | 98.18% | 97.92% | -0.25% |
| *Staphylococcus saprophyticus*  outlier GeneID 3615360, score 40.4  COG0199, “30s ribosomal protein s14” | 9.23% | 9.19% | 9.27% | 8.88·10^-16^ | 98.22% | 98.48% | 0.26% |

Text S2, Table 6. Robustness to selection acting on a non-coding DNA.

**Text S2, Table 6.** A test for robustness of the Random Forest (RF) classifier‐based methodology with regard to selection acting on non-coding DNA (ncDNA). The „original run“ columns represents the data used throught the paper. The „repeated run“ means the RF-based computational procedure for detecting OCU genes was run again on these 10 genomes with no changes in the input parameters except the 'seed' for initializing the random number generator; this was done to quantify variation introduced by our methodology for OCU detection. The „masked 20 nt upstream“ are the datasets where 20 nucleotides upstream of translation start codon were not taken into consideration when computing mono- and di-nucleotide frequencies in the ncDNA; this region of prokaryotic ncDNA was found to be under much stronger selective pressures than the rest of the ncDNA [1].

|  | % OCU in genome | | | *p*-value for difference in AUC scores (sign test) | agreement with original run | | |
| --- | --- | --- | --- | --- | --- | --- | --- |
| organism | original run | repeated run | masked 20 nt upstream |  | repeated run | masked 20 nt upstream | difference |
| *Bacillus subtilis* | 8.44% | 8.68% | 9.54% | 8.88·10^-16^ | 98.24% | 96.43% | -1.81% |
| *Borrelia burgdorferi* | 21.97% | 21.60% | 23.48% | 8.88·10^-16^ | 93.71% | 90.68% | -3.03% |
| *Deinococcus radiodurans* | 10.85% | 11.01% | 10.82% | 8.88·10^-16^ | 97.64% | 96.09% | -1.55% |
| *Escherichia coli* K12 | 7.49% | 7.41% | 7.82% | 8.88·10^-16^ | 98.49% | 97.73% | -0.77% |
| *Halobacterium sp.* | 19.24% | 18.95% | 19.28% | 8.88·10^-16^ | 94.35% | 92.10% | -2.26% |
| *Helicobacter pylori* 26695 | 19.18% | 19.66% | 20.00% | 8.88·10^-16^ | 95.71% | 93.20% | -2.52% |
| *Mycoplasma genitalium* | 16.34% | 17.63% | 19.57% | 8.88·10^-16^ | 94.84% | 90.75% | -4.09% |
| *Pelagibacter ubique* HTCC1062 | 19.12% | 19.43% | 19.43% | 8.88·10^-16^ | 95.36% | 93.19% | -2.17% |
| *Pseudomonas aeruginosa* | 6.19% | 6.19% | 6.12% | 8.88·10^-16^ | 98.64% | 98.12% | -0.52% |
| *Streptomyces coelicolor* | 6.87% | 6.72% | 7.03% | 8.88·10^-16^ | 98.46% | 97.79% | -0.67% |

**Reference:**

[1] Molina N, van Nimwegen E (2008) Universal patterns of purifying selection at noncoding positions in bacteria. Genome Res 18: 148-160.
